# Prioritising Mangrove Ecosystem Services Results in Spatially Variable Management Priorities

**DOI:** 10.1371/journal.pone.0151992

**Published:** 2016-03-23

**Authors:** Scott C. Atkinson, Stacy D. Jupiter, Vanessa M. Adams, J. Carter Ingram, Siddharth Narayan, Carissa J. Klein, Hugh P. Possingham

**Affiliations:** 1 ARC Centre of Excellence for Environmental Decisions, Centre for Biodiversity and Conservation Science, School of Biological Sciences, The University of Queensland, St. Lucia, Australia; 2 Wildlife Conservation Society, Melanesia Program, Suva, Fiji; 3 Wildlife Conservation Society, Global Conservation Program, New York City, United States of America; 4 National Center for Ecological Analysis and Synthesis (NCEAS), University of California at Santa Barbara, Santa Barbara, United States of America; 5 School of Geography, Planning and Environmental Management, The University of Queensland, St. Lucia, Australia; Griffith University, AUSTRALIA

## Abstract

Incorporating the values of the services that ecosystems provide into decision making is becoming increasingly common in nature conservation and resource management policies, both locally and globally. Yet with limited funds for conservation of threatened species and ecosystems there is a desire to identify priority areas where investment efficiently conserves multiple ecosystem services. We mapped four mangrove ecosystems services (coastal protection, fisheries, biodiversity, and carbon storage) across Fiji. Using a cost-effectiveness analysis, we prioritised mangrove areas for each service, where the effectiveness was a function of the benefits provided to the local communities, and the costs were associated with restricting specific uses of mangroves. We demonstrate that, although priority mangrove areas (top 20%) for each service can be managed at relatively low opportunity costs (ranging from 4.5 to 11.3% of overall opportunity costs), prioritising for a single service yields relatively low co-benefits due to limited geographical overlap with priority areas for other services. None-the-less, prioritisation of mangrove areas provides greater overlap of benefits than if sites were selected randomly for most ecosystem services. We discuss deficiencies in the mapping of ecosystems services in data poor regions and how this may impact upon the equity of managing mangroves for particular services across the urban-rural divide in developing countries. Finally we discuss how our maps may aid decision-makers to direct funding for mangrove management from various sources to localities that best meet funding objectives, as well as how this knowledge can aid in creating a national mangrove zoning scheme.

## Introduction

Mangroves provide important provisioning (e.g., timber and food, including fisheries production), regulating (e.g., climate regulation, water purification, coastal protection, erosion control), cultural (e.g., recreation, aesthetic value, spiritual value), and supporting (e.g., nutrient cycling) services to millions of coastal residents in tropical and subtropical latitudes around the globe [1–4]. These services are critically important in Pacific Island states where high proportions of the population are heavily dependent on mangrove resources for subsistence and livelihoods [5,6]. Globally, tidal marsh and mangrove ecosystem services (ES) are valued at approximately US$32 billion annually, which translates to approximately US$194,000 ha^-1^ yr^-1^ [7]. A regional valuation from the Pacific Islands suggests the composite value of mangroves across multiple services ranges between US$4,300-$8,500 ha^-1^ yr^-1^ [8], which represent significant market values when considered alongside mean annual household incomes per-adult-equivalent in the region (e.g., for Fiji, US$2,664 in 2008; [9]). In addition, other non-market benefits (e.g., cultural and aesthetic values) provided by mangroves cannot easily be monetised, but should be considered for decision making related to mangrove management [10].

Despite the many values associated with mangroves, worldwide rates of mangrove loss have been high and are accelerating [1,11,12]. Approximately 20–35% of mangrove area has been cleared since 1980 [13], largely to accommodate coastal development and aquaculture. The Pacific may experience a further 13% loss of existing mangrove area (~524,000 ha) by 2100 because of sea level rise [14], which will put greater pressure on remaining mangrove resources. Yet, decisions to clear remaining mangroves rarely take into consideration the lost market and non-market value of their ecosystem services [15].

Following recent large-scale natural disasters, such as the Indian Ocean tsunami in 2004, Hurricane Katrina in 2005, and Typhoon Haiyan in 2013, there has been increasing momentum in the scientific literature and policy arenas to recognise the services that coastal wetlands—including mangroves—provide for coastal protection, in terms of reduced flood risk, infrastructure damage and human injury and mortality [16–18]. While there is debate about the extent to which mangroves can provide protection from large tsunamis and tropical cyclones [19–22], there is evidence that mangrove stands with certain characteristics can mitigate impacts of storm surges [23] and potentially smaller tsunami waves [24]. Consequently, many donors, agencies and non-governmental organisations have been promoting the value of mangroves for coastal protection and resilience [25–27]. Evidence for this interest comes from a surge in climate adaptation financing, with the aspiration that areas managed or restored to provide this service will also provide co-benefits (e.g., for biodiversity conservation, fisheries, tourism and recreation; [18,28,29]). These co-benefits may arise when the mangrove stands with the highest values for coastal protection spatially overlap with areas that provide high values for other ecosystem services.

There is reason to suspect that multiple ES benefits from mangrove protection may be rare. Coastal wetlands that provide high values for coastal protection in areas directly adjacent to urban and peri-urban human settlements and highly altered watersheds are likely to have degraded biodiversity and fisheries services due to heavy anthropogenic impacts from: pollutants in run-off (e.g., sediments, nutrients, chemicals, acid sulphate soils); dredging; selective deforestation and fragmentation; over-harvesting; and hydrological alteration (e.g., [30,31]). For example, loss of catchment forest cover has been associated with marked reductions in fish species richness in lower river reaches (including mangrove estuaries; [32]). Further, dense human settlement may result in heavy harvesting of mangrove macro-invertebrate fauna that can affect overall food web structure, including benthic and pelagic fisheries, while potentially negatively impacting mangrove forest ecosystem processes (e.g., nutrient and nitrogen cycling) that support biodiversity and ecosystem functions [33]. Difficulties in identifying conservation priorities and incentivising people to conserve nature often relate to the importance and challenges of understanding trade-offs in ES provisioning and identifying where and to whom the accrual of benefits associated with different services occurs. For instance, the beneficiaries of coastal protection typically reside close to where to the service is produced, whereas biodiversity benefits may be enjoyed by tourists visiting a location far from where they live.

It is necessary to understand how the provisioning and values of different mangrove ES vary spatially in order to prioritise mangroves for management and identify possible trade-offs and synergies in achieving different priorities given limited resources for mangrove conservation. The aims of this study were therefore to: (1) identify spatial priorities for optimising four separate ES objectives of mangrove management (coastal protection, fisheries, carbon storage, and biodiversity); and (2) identify and explore spatially explicit synergies and trade-offs of in optimising these four ES objectives at priority sites. We chose Fiji as a case study to demonstrate an application of ES mapping in a relatively data-poor context, and also because there are potential policy levers to incorporate mangrove ecosystem service values into decision-making processes regarding coastal development approvals. We discuss the implications of spatial variability in mangrove ecosystem service provisioning with regard to resource allocation for protection and management, as well as opportunities to incorporate our results into national policy frameworks that guide decision-making for coastal management.

## Methods

### Study area

**Fig 1:**
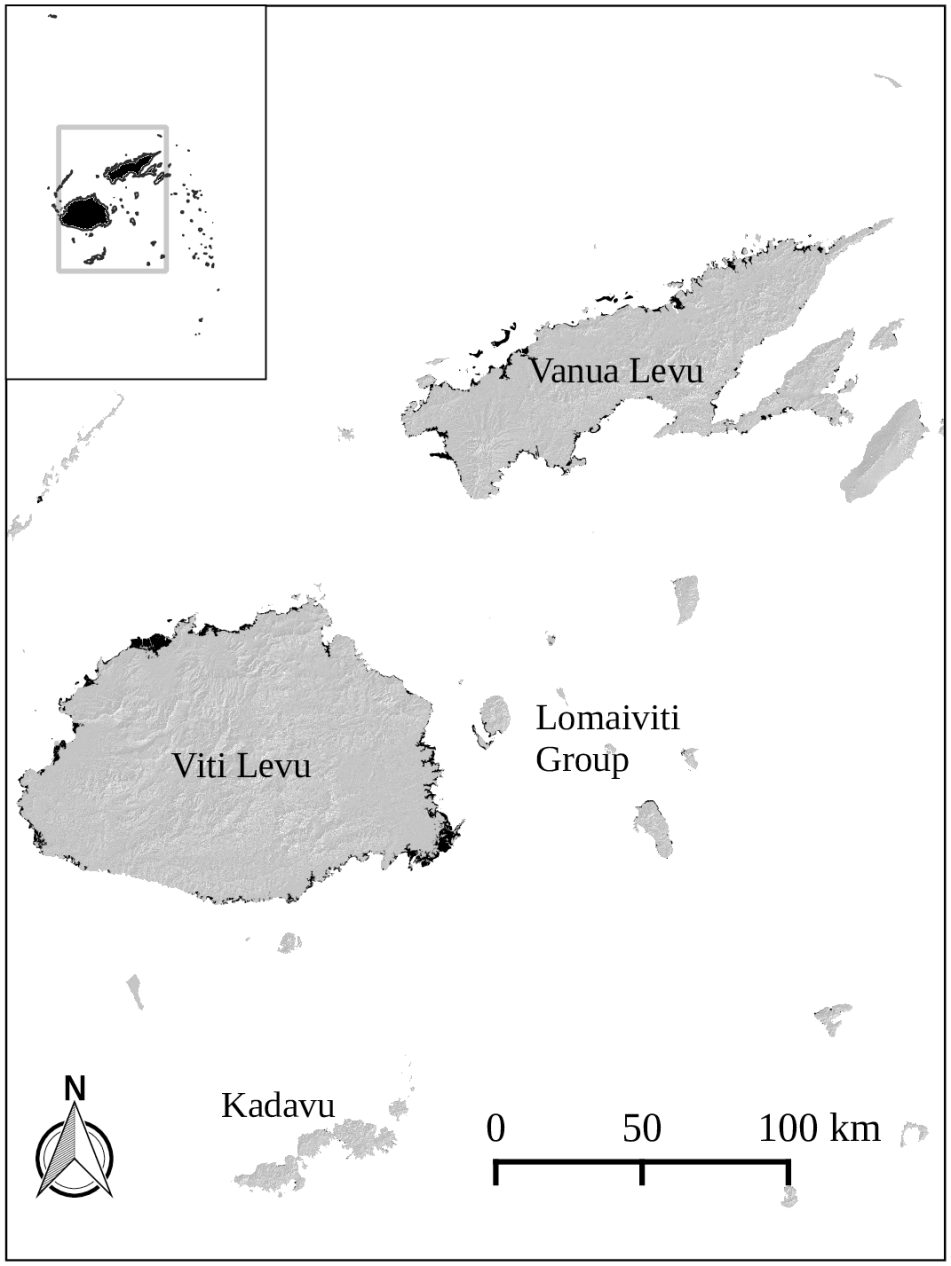
Central areas of the Fijian Archipelago (inset). Greater than 95% of Fiji's mangrove areas occur on the islands of Viti Levu, Vanua Levu, Kadavu, and within the Lomaiviti Group. Dark polygons are mangrove areas mapped in this study.

The study area encompasses the central core of the Fijian archipelago (Fig 1). Of Fiji’s approximately 49,300 ha of mangrove forest, more than 47,000 ha (95.5%) occur on the coasts and reef islands of Fiji’s two largest islands, Viti Levu and Vanua Levu, with much of the remainder occurring on the islands of the Lomaiviti and Kadavu provinces. To derive maps of mangrove areas, we combined a recently released global mangrove dataset [34] with habitat maps digitised by the Fiji Department of Forestry using 2001 Landsat Enhanced Thematic Mapper Plus (ETM+) satellite imagery (S1 Table). Including the global dataset allowed us to overcome significant omissions and errors in the in-country habitat maps, while still allowing for the in-country maps’ greater accuracy in certain locations. Importantly, incorporating the global dataset allowed us to carry out a nearly countrywide assessment of the spatial variation in mangrove ES provision.

### Planning units

We chose to use contiguous mapped mangroves as planning units (PUs) for our prioritisation, with the exception of several large and laterally spread out mangrove areas which were split into multiple planning units along geographic features delineating boundaries, such as where large rivers bisected large mangrove areas or where small corridors connected larger clumps of mangrove (S1 Fig). We mapped 1,133 individual mangrove areas, with sizes ranging from 0.04 ha to 3,387 ha (median 5.6 ha). To derive planning units from the mapped mangrove areas we chose to buffer our mangrove areas by 500 m (included within the PU; see S1 Table) in order to capture the flow of services to local communities (defined here as living less than half a kilometre away from the mangrove). This allowed us to capture both benefits of the ES provided to the local population (e.g., fisheries production) and costs associated with prohibiting certain uses of a mangrove (e.g., lost lease revenue or firewood extraction). While this assumption does not account for the longer distances Fijians will travel to access particular stands of mangroves, particularly if within their land tenure or traditional fisheries areas, available data does not allow for better estimates of local populations' usage of mangrove areas. Where mangroves had clearly defined ocean frontages we attributed to that PU the area of coral reef within a 10 km buffer to capture benefits to nearby reef fisheries attributed to greater connectivity between mangrove and coral reef ecosystems [35]. Planning unit sizes ranged from 5.2 ha to 5,568.6 ha (median = 101.3 ha).

### Mangrove management scenarios

We explored four ES provided by mangroves which decision makers are interested in prioritising for management: coastal protection, fisheries, biodiversity, and carbon storage [2], and consider the spatial variation of the benefits and costs associated with managing these services across Fiji, the extents of which neither production nor value have previously been explored. Unless explicitly stated otherwise, we use the term 'cost' in reference to opportunity costs associated with managing for a particular ES exclusively, and not inclusive of any costs associated with management, such as monitoring.

**Fig 2:**
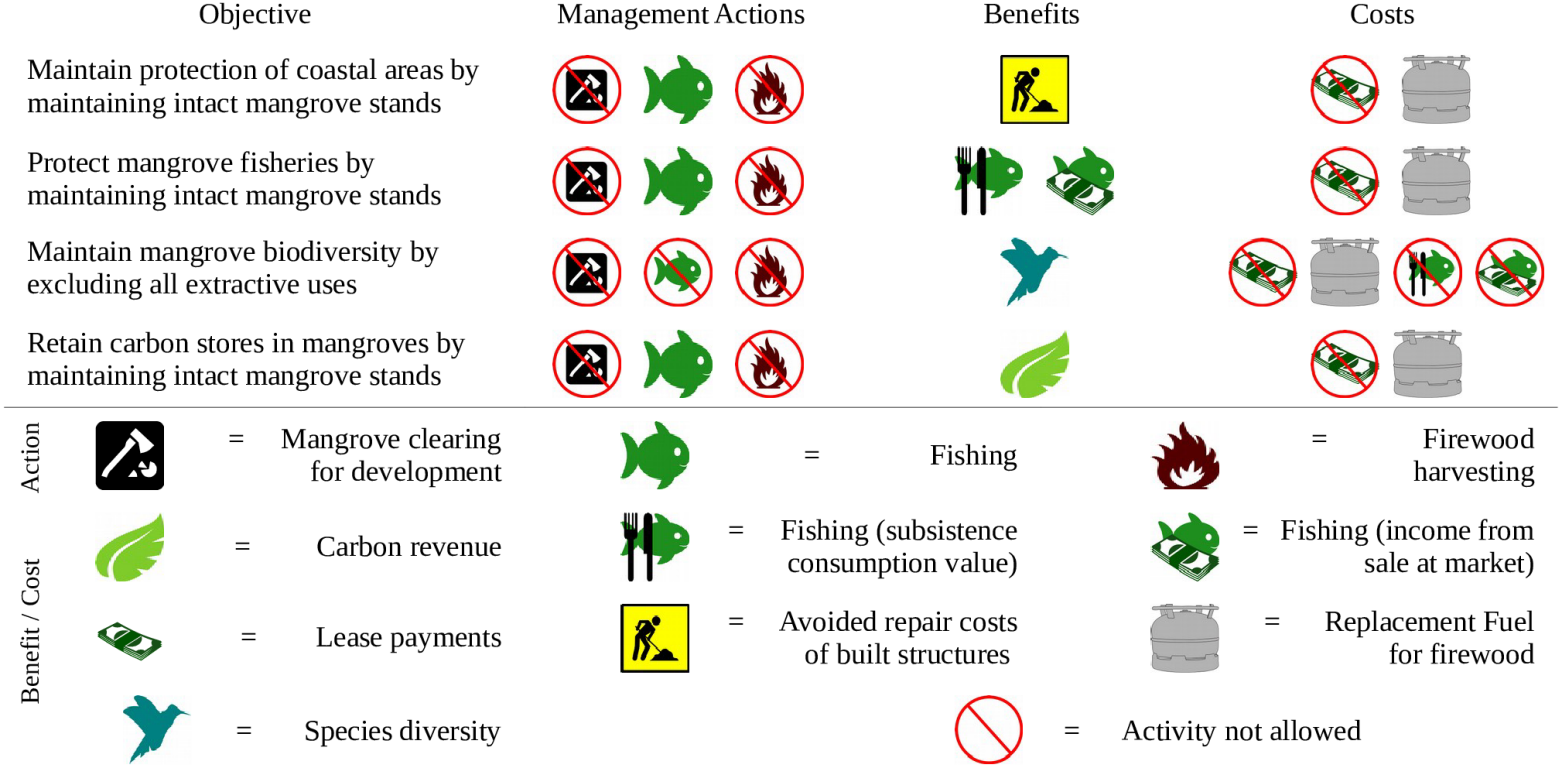
Management objectives and associated mangrove management actions aimed at ensuring long-term provisioning of the ES.

For each ES we: (1) set the objective of managing for a particular ES (Fig 2); (2) identified associated management actions; (3) defined the benefits and opportunity-costs associated with management actions ensuring the long-term provisioning of the ES being provided; (4) collated available data associated with each ES in Fiji, or used regional data if Fiji-specific data were unavailable; (5) estimated the available quantity of the ES provided by individual mangrove areas within geographic information system (GIS) software; (6) calculated (where possible) the spatially explicit monetary value of both benefits and opportunity costs; and (7) conducted a cost-effectiveness analysis to identify priority sites for managing for each ES (e.g., see [36]). We describe each of these steps and data sources in more detail below.

### Ecosystem Service Benefits and Costs

We calculated the costs and benefits associated with different management scenarios—on a per hectare basis—using a benefit-transfers approach to estimate the quantity of service available [37,38], based on both literature- and government-sourced ES provisioning (e.g., the amount provided) and economic values (e.g. the monetary value) from Fiji or neighbouring islands where possible. We first estimated the total biophysical quantity provided of each ecosystem service (e.g., percentage of wave attenuated, tonnes of carbon stored). Where possible we then calculated a monetary value associated with the ES. Costs associated with management were estimated based on the opportunity costs associated with the management action [39]; for example, protecting a mangrove for biodiversity would exclude all extractive uses and therefore incur opportunity costs for coastal development (estimated based on current leases), timber extraction for firewood (estimated based on the replacement cost of alternative fuel sources) and fishing for consumption and marketable fishery products (estimated based on 2015 fish market prices). Costs were based on the most recent reported Fijian prices for the goods and services associated with the above opportunity costs. Equations for all cost and benefits and associated assumption and data sources are shown in Table 1.

**Table 1:** Parameters used in calculation of costs and benefits for four mangrove ecosystem services.

Variable	Sym.	Equation used	Assumptions made	Data inputs	Source
***Benefit calculations***					
Wave attenuation	*w*	*w* = -8e^-7^*x*^2^+0.0016*x*+0.128 where *x* = width of mangrove at point of interest	Proportionate change in wave height as a function of mangrove width. No account of species specificity or mangrove density impacts on attenuation.	Depth of mangrove (m). Wave attenuation model.	[40]
Replacement costs of roads	*r*	Roadway length × Repair costs per km	Converted from year 2000 USD. Based on global average; Fiji specific costs will differ.	Road network layer. Inundation zone layer. Road repair costs.	[41]
Replacement cost of trams	*t*	Length of tramline × Repair cost per km	Repair costs $23,504 km^-1^	Tram lines layer. Repair costs. At-risk inundation zone layer.	[42]
Replacement cost of buildings	*b*	Estimated # of residential buildings × Value of residential buildings	Total building value is lost. All buildings are residential	Buildings/villages layers. Replacement value by district. Estimated buildings layer. At-risk inundation zone layer.	[43,44]
Species	*S*	*S = c A*^*z*^	Non-linear increase of mangrove associated species with increasing area; where *c* is a constant and equals the number of species expected if one unit (e.g. 1 ha) of mangrove were present, and z is the rate (or slope) of the accumulation of new species as area increases. We assume *c* = 10; *z* = 0.3	List of mangrove associated floral and faunal species in Fiji	[45,46]
Carbon storage	γ	CO_2_e stock × % of stock releasable × Decay rate of releasable stock × Market price of carbon	Uniform CO_2_e stocks (t ha^-1^) in a mangrove. Does not account for different carbon storage and sequestration values per species, forest density, or local geomorphology	Mangrove layer. Carbon storage estimates. Price of carbon.	[8]
Percent coral cover	*c*	Area of coral reef within 10 km ÷ Ocean area within 10 km	Higher coral reef density in the nearby seascape provides greater fishery benefit. Seascape areas within 10 km are ocean areas within 10km of a mangrove's front, accounting for land barriers to marine movement and connectivity (Supporting Information)	Coral reefs layer. 10 km distance from mangrove layer.	Fiji Department of Lands
***Cost calculations***					
Timber costs	*T*	Firewood burn-time required/provided by the mangrove × Replacement fuel consumption × Fuel replacement price	Mangrove firewood production: 511 kg ha^-1^ yr^-1^. Each household in the PU requires enough timber for 1 cooked meal every day (1 hour). Demand on mangrove is limited by total production. 3.56 kg firewood per meal. Kerosene price: $0.61 per litre	Mangrove layer. Cooking/fuel conversion. Kerosene price. # households in PU	[8,47,48]
Lease Cost	*L*	Potential leases × Regional lease values (per lease)	Average foreshore lease 200 m along shore × 100m inland (2 ha). If no mapped ocean front, lease footprint is 4 ha. Lease values derived from annual foreshore lease data provided by the Ministry of Lands and Mineral Resources.	Mangrove layer. Mangrove front layer. Lease values.	Fiji Department of Lands
***Both benefit and cost calculations***					
Fisheries value—market	*f*_*m*_	{(Biological production of fishery − Subsistence consumption) × Market value} − {Return distance to market × Price of fuel × Fuel consumption}	Subsistence values are prioritised over market values. Excess production only can be sold. Weekly market sale of five fish. Fish sold at the nearest market’s average price. Fuel cost: $0.91 l^-1^. Fuel consumption @ 1 litre per 10 km along roadways. For markets not reachable by road, fuel cost is higher and market price lower	*#* of households in PU. Mangrove area layer. Fish market values. Return travel distance to market.	[47,49]
Pollution penalty	*p*	$P=\frac{\left(b-a\right)\left(P-min\left(P\right)\right)}{max\left(P\right)-min\left(P\right)}+a,$ where *b* = 0.5 (more polluted), and *a* = 1 (less polluted)	Fishery productivity reduced by run-off pollution. Pollution run-off is a function of forest cover in a catchment.	Modelled maximum pollution loads by catchment. Forest cover layer.	[36,50]

PU = planning unit; CO_2_e = carbon dioxide equivalent. Groupings indicate whether the variable contributes to benefit calculations, cost calculations, or to both.

All benefits and costs were calculated as 10-year present values, assuming a 3% discount rate. We used a 3% discount rate to coincide with seawall construction estimates [51]. The spatially explicit ES values provided by individual mangrove areas were determined from layers built in a GIS which we derived from a variety of available sources (See S1 Table for full details). All GIS analyses were carried out using QGIS 2.10 [52] and GRASS GIS 6.4 [53].

### Cost-effectiveness prioritisation

We calculated the benefit-to-cost ratio (BCR) for each ES by developing benefit- and cost-functions for each service based on spatially estimated quantities of a service. BCR calculations are presented below. Unless noted otherwise, each benefit term (the numerator) was calculated as the estimated total value, converted into 2015 US Dollars. Complete methods used to derive spatial layers and estimations of parameters are presented in S1 Table. It is worth noting that our use of the BCR was for cost-efficient prioritisation of sites for each management objective and not for use in a classical economic cost-benefit analysis benefit-cost analysis (where actions are taken if benefits outweigh costs). Thus, sites with ratios less than one might still be prioritised for management if they were highly ranked.

#### Coastal protection

In the absence of measuring or modelling the functional form of the relationship between mangrove wave attenuation and reduced damage to built structures in Fiji, we assume that changes in wave height result in proportionate reductions in damage to structures near the coastline. We calculated the BCR of managing mangroves for coastal protection (*CP*) from large tropical storm waves as:

$$CP=\frac{w_{b}b+w_{r}r+w_{t}t}{L+T}$$

where *w* is the mean proportionate wave reduction performance (e.g., [40]) provided by the mangrove at associated buildings (*b)*, roads (*r*), and sugar cane tramways (*t*) located within the sub-10 metres above mean sea level (mamsl) inundation zone adjacent to and protected by the mangrove, where *b*, *r*, and *t* are the replacement values of buildings, roads, and tramways being protected, and, *L* and *T* represent opportunity costs associated with foreshore development leases and timber (firewood) harvesting, respectively (Table 1). We assume that all firewood collection is prohibited; however, sustainable collection could in theory be allowed without detrimental impact on service provisioning [40]. Although not calculated here due to insufficient data, mangroves have also been observed to provide maintenance benefits for land-ward seawalls [54].

#### Fisheries

We calculated the BCR of managing for fisheries services as:

$$F=\frac{\left(f_{s}+f_{m}\right)*p*c}{L+T}$$

where the total fishery value (*F*) is a function of fish catch and crustacean harvest values for subsistence (*f*_*s*_) and sale at market (*f*_*m*_*)*, the quality and quantity of fish is positively impacted by greater coral reef cover adjacent to the mangrove (percent coral cover, *c*), and run-off pollution negatively impacts upon fishery production and value (pollution penalty index, *p*). Costs, *L* and *T*, remain the same as above. The values of *f*_*s*,*m*_ incorporated estimated protein demands of the local population, fish market prices (as of March 2015; S2 Table), and estimated travel costs to the nearest major fish market via the road network (see S1 Table).

#### Biodiversity

We calculated the BCR of managing for biodiversity (*BD*) services as:

$$BD=\frac{S}{L+T+\left(f_{s}+f_{m}\right)}$$

where *S* is the estimated number of species present in a mangrove based on a non-linear species-area curve, and costs are similar to above, with the addition of the combined total benefits of both subsistence and market fisheries (*f*_*s*_ *+ f*_*m*_) as a cost, assuming that fishing and harvesting activities are prohibited when a mangrove is managed for biodiversity. We base *S* on available data of mangrove associated species in Fiji [45], which includes mangrove species, common and uncommon floral mangrove associates, and both fish and invertebrate marine species.

#### Carbon storage

We calculated the BCR of carbon storage and sequestration (CB) as:

$$CB=\frac{A_{m}*\gamma *CER_{\$}}{L+T}$$

where *A*_*m*_ is the area of the individual mangrove stand, and *γ* is a conversion factor for estimating the carbon storage value of the mangrove; costs *L* and *T* are the same as above. The value of *γ* assumes that carbon is stored in a mangrove at a constant rate of 900 t CO_2_e ha^-1^; further, 60% of that is assumed to be potentially releasable if the mangrove were cleared, of which 45% could decay annually [8]. We calculated the present value of stored carbon at the current Certified Emission Reduction CO_2_e unit price, *CER*_*$*_ (EUR €0.64, as of 24 November 2015), converted to US Dollars. Carbon storage in mangrove systems will vary according to the species present, tree densities, and sediment characteristics [4]; however, such data was not available across the study area.

### Cost and benefit correlations

To better understand potential primary drivers of spatial variation within the mangrove provisioning of ES across Fiji, we conducted correlation analyses of the primary inputs into our BCR calculations, as well as between final overall costs, benefits, and ranks (S2–S5 Figs).

#### Priority mangroves

Each mangrove unit was ranked from highest to lowest BCR for each of the respective ES we considered. The top ranked mangrove areas for each ES, comprising up to 20% of the total mangrove area in Fiji (hereafter referred to as priority areas), were mapped. In addition, we display the benefits that can be achieved by managing these priority areas for each ES relative to the percent of total opportunity costs associated with managing mangroves for the service.

## Results

**Fig 3:**
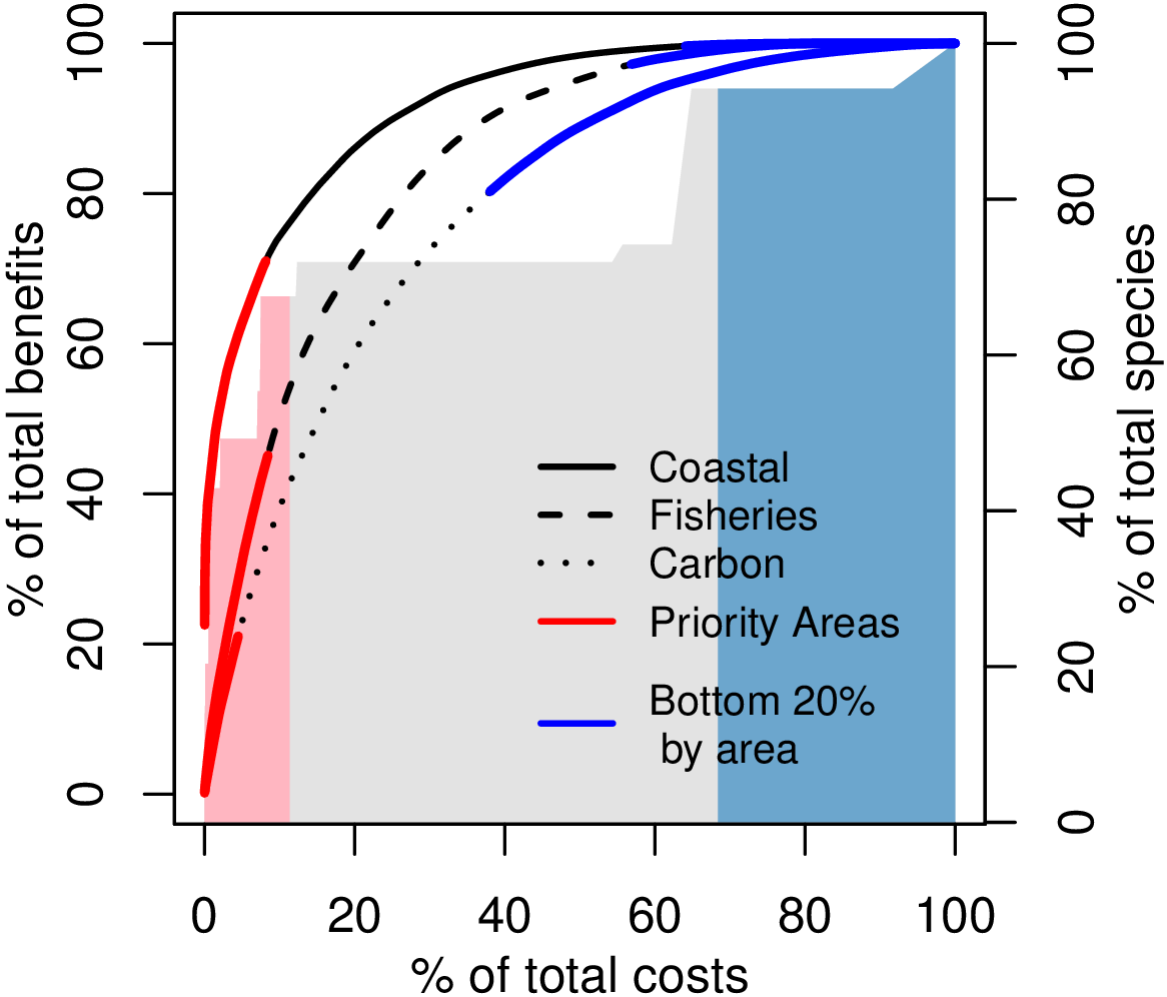
Percent of total cumulative ES benefits versus the percent of overall opportunity costs when managing each mangrove ES service objective; red and blue lines indicate the priority and bottom 20%-by-area for each service. Coloured polygons indicate the cumulative percent of the total number of mangrove associated species that would be protected by managing sites in order of their priority ranking.

The results of the mapping of both overall ES provisioning and economic value for each mangrove area in Fiji can be found in S6–S9 Figs. Coastal protection benefits were highest in areas of concentrated infrastructure and high flooding risk, while fisheries benefits represent a trade-off between human usage and the resultant pollution associated with human development. Both carbon storage and biodiversity ES have overall benefits that are a function of area, but priority rankings driven by their low overall monetary values relative to management costs, placing priority areas away from populated areas (carbon storage) or to the smallest mangrove areas (biodiversity). For all services, managing priority areas provided the most efficient means to accumulate overall ES benefit versus lost opportunity costs associated with management (Fig 3). Unless otherwise noted all monetary values are in 2015 US Dollars.

### Coastal protection

**Fig 4:**
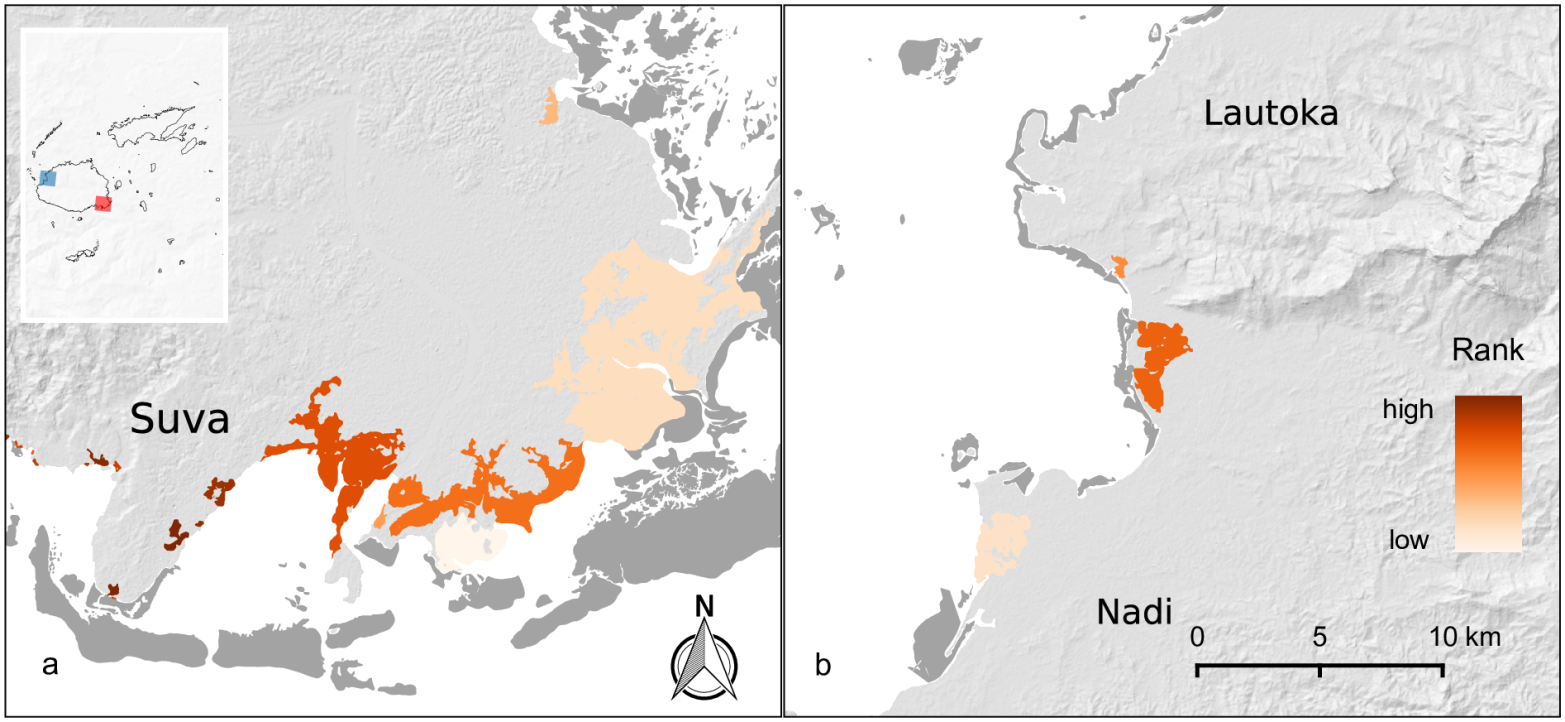
Ranking of priority sites for coastal protection services. Panel (a) (red inset polygon) centres on the greater Suva area; the darkest coloured areas are the highest ranked priority sites in Fiji. Panel (b) (blue inset polygon) centres on the Nadi and Lautoka area in the Western Division; populations are also high in the area, but many areas where mangroves may have existed have been cleared for coastal and resort development. Little development occurs behind extant mangroves in this area. Dark grey areas are corals reefs.

Calculated on a per hectare basis, coastal protection values ranged from $88–$6.1M ha^-1^ over 10 years (mean = $51,870; sd = $291,469). Priority mangroves for coastal protection services (53 mangrove areas; median size 33.8 ha) were generally smaller than priority areas for fisheries and carbon services, and were located principally around the populated greater-Suva and Rewa Delta areas, and to a lesser extent around the cities in Fiji’s Western Division (Nadi and Lautoka, Fig 4). The total benefit of coastal protection per mangrove unit was weakly correlated with regional variations in average building replacement costs (*r* = 0.26; *p* < 0.001); notably however, the top fourteen (14) priority mangrove areas were found around Suva where population is both greater and mean building replacement values are higher (~$87,610) than the rest of Viti Levu ($20,525) or Fiji as a whole ($15,663; S3 Table). Costs (10-year net-present-value (NPV)) per hectare (range $50 –$33,451 ha^-1^; mean = $1,723 sd = $3,004) were driven primarily though lost annual development lease payments (*r* = 0.99, *p* < 0.001; S2 Fig), which around Suva were among the lowest on Viti Levu (~$240). Managing coastal protection priority mangroves maintains 71% of the overall coastal protection benefits provided by mangroves at less than 9% of total opportunity costs (Fig 3).

### Fisheries

**Fig 5:**
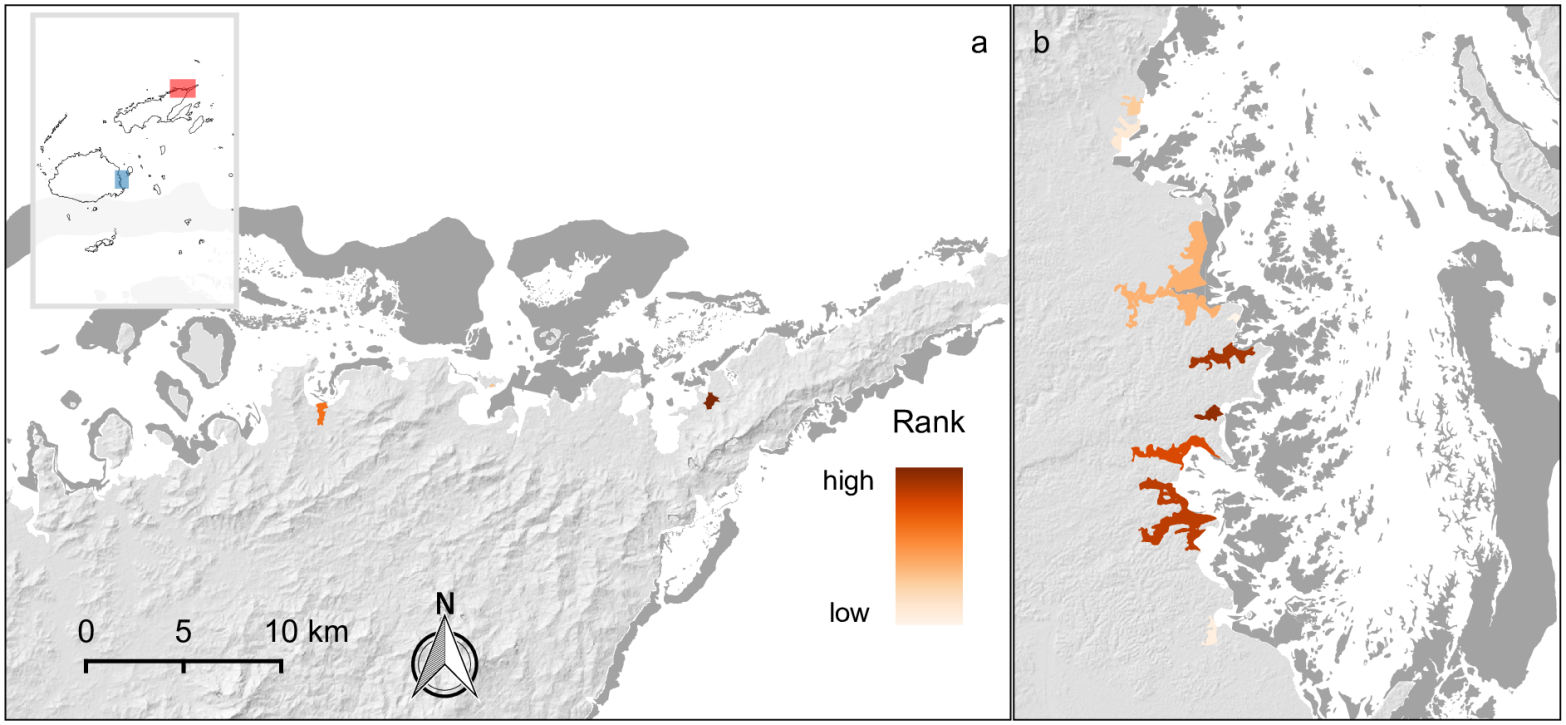
Priority sites for fisheries services. The highest ranked priority site was found on north-eastern Vanua Levu (panel a, red inset polygon) but a large clumping of the highest ranking priority sites occurred north of the Rewa Delta (panel b, blue inset polygon) on eastern Viti Levu. Dark grey areas are coral reefs.

Overall fisheries benefits were greatest in areas where population is higher, but where run-off pollution is low, with 10-year NPV per hectare ranging from $0 –$3,839 ha^-1^ (mean = $455; sd = $595). Fiji's most populated and developed areas coincide with river catchments areas where large amounts of land clearing have historically occurred, forest cover is lower, and estimated run-off pollution is high (S10 Fig). Pollution was weakly negatively correlated to the number of households in a PU (*r* = -0.11; *p* < 0.001) and both subsistence and market fish values (*r* = -0.16; *p* < 0.001; *r* = -0.13; *p* < 0.001, respectively; S3 Fig). Priority mangroves represented a trade-off between the spatial co-occurrence of higher population, higher subsistence and market fishery values, and pollution penalties. Priorities (40 mangrove areas; median size 58.0 ha) occurred where fisheries benefits were of moderate value and were in catchments with lower pollution penalties, for example, around the north-east of Vanua Levu (Fig 5a) and east of Viti Levu. Proximity of dense coral reef areas is weakly correlated with overall fisheries benefit (*r* = 0.25; *p* < 0.001); however, clustering of four of the top five priority sites on Viti Levu’s eastern coastline (Fig 5b) indicates the influence of high densities of nearby coral reefs, access to a high value fish market (Suva), and avoidance of pollution. Priority mangroves maintain nearly 47% of total fisheries benefit at less than 9% of the total overall opportunity costs of management (Fig 3).

### Biodiversity

**Fig 6:**
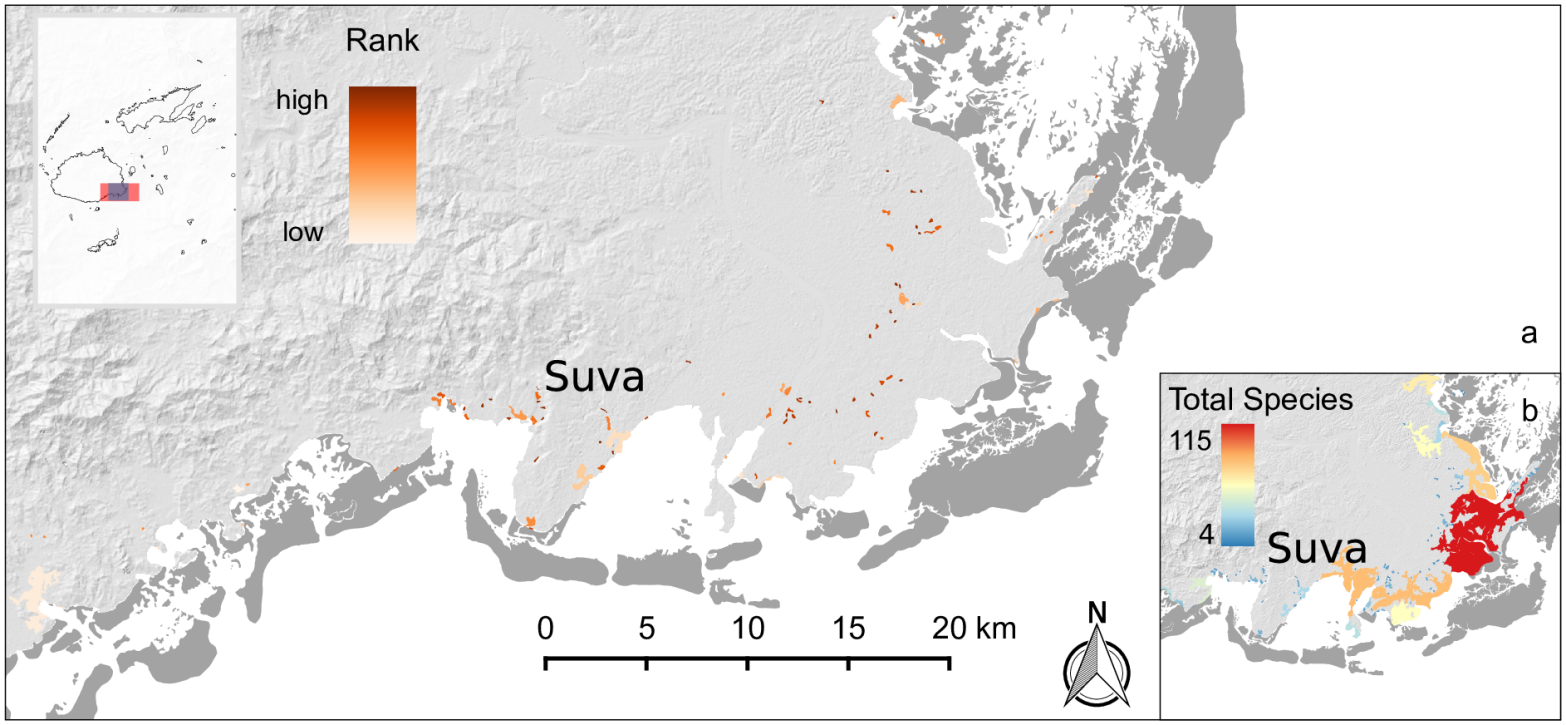
Priority sites for biodiversity services. The highest ranked priority mangrove for biodiversity was actually within the town limits of Suva and was the second smallest mangrove stand mapped (530 m^2^; panel a, red inset polygon). Priority mangroves contrast greatly with mangroves having the highest overall biodiversity benefit (*S*), for example, around the Rewa Delta area (panel b, blue inset polygon). Dark grey areas are coral reefs.

Median size of priority mangroves for managing biodiversity was the smallest among all ES we considered (~ 3.5 ha; 852 mangroves). We estimated that 67.5% of the total number of mangrove associated species (~77 species) would be protected by managing priority sites, at just over 11% of the total opportunity costs associated with management (Fig 3). Priority ranking of mangroves for biodiversity services was most strongly correlated with costs associated with foregone fisheries benefits (assuming fishing has been disallowed; *r* = 1.00; *p* < 0.001; S4 Fig). Priority mangroves were selected primarily though minimisation of opportunity cost, resulting in priority mangroves being very small in area, and often those without direct ocean frontage (e.g., avoidance of fishing costs) where overall benefits are small (Fig 6a and 6b).

### Carbon storage/sequestration

**Fig 7:**
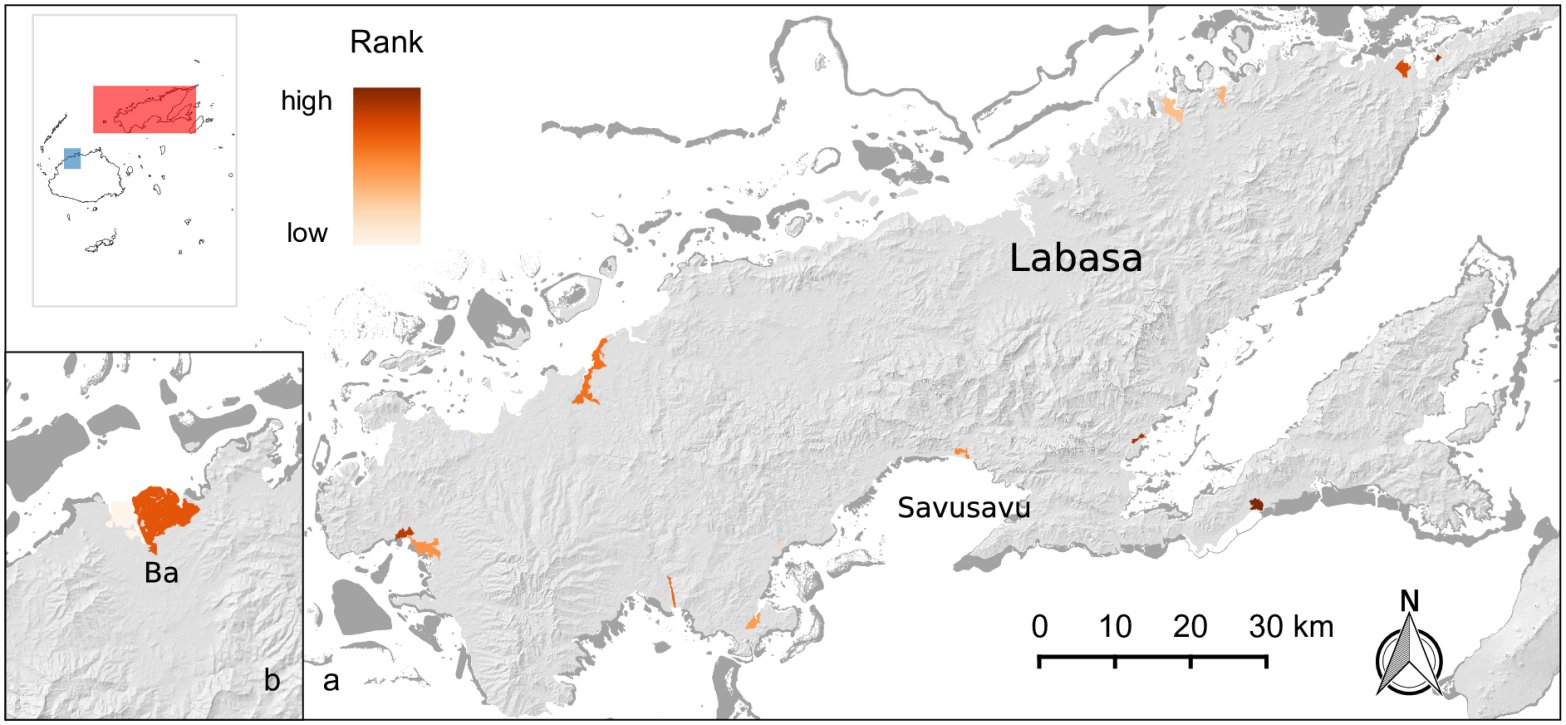
Carbon storage priority mangroves. The majority of the 19 priority areas were on the island of Vanua Levu, where population and development is generally lower (panel a, red inset polygon). Several large priority areas are found on Viti Levu, however; for example, around the large mangrove stands near the mouth of the Ba river (panel b, blue inset polygon). Dark grey areas are coral reefs.

Carbon storage benefits were assumed to be a linear function of mangrove area. Timber/firewood costs (*T*) alone were significantly, weakly correlated to carbon storage BCR (*r* = 0.32; *p* < 0.001; S5 Fig). The median size of the priority mangrove areas (19 areas) was 196.2 ha. Benefit-cost ratios for carbon storage services were the lowest for all the monetised ES benefits we considered due to low prices of carbon futures (EUR €0.64 (USD $0.68) at time of analysis; www.theice.com), assuming a carbon market were in place. Differentiation between overall mangrove carbon storage values was seen where there were trade-offs between greater carbon sequestering benefits (very large mangroves areas) and higher lease (e.g., longer mangrove fronts and regional lease differences) and forestry product replacement costs (proximity to populated areas). Most (14 of 19 areas) priority mangrove areas for carbon storage were found on Vanua Levu (Fig 7a), where populations, and therefore costs, are generally lower, with notable exceptions of two large mangrove areas on Viti Levu (Fig 7b). Priority mangroves provided over 21% of the overall carbon storage benefits at 4.5% of the overall opportunity costs of management (Fig 3).

### Co-benefits

Co-benefits of managing for a particular ES are shown in Table 2 as a percentage of the overall priority area for the service. For example, 42.6% of priority areas for coastal protection services are also priority areas for fisheries services. The small disparity when fishery priority areas are also priorities for coastal protection (41.8% by area) is a result of different mangroves contributing to priority designation (e.g., ~20% by area) for each ES, which results in slightly different total areas being selected. If sites are selected randomly (assuming there is no correlation between ES) one would expect only 4% overlap in priority areas; the 20% of priority sites selected for one ES should only select 20% of the priority sites for another ES, on average (resulting in 4% overlap). No individual mangrove areas were priorities for all four services. When managing for either of coastal protection, fisheries, or carbon storage services approximately 39–48% by area of priority mangroves were also priorities for the other two services; co-benefits for biodiversity were much lower.

**Table 2:** Percent by area of co-benefits when priority areas are managed for an ES.

	CP	F	BD	CB
CP	—	42.6	16.1	47.7
F	41.8	—	0.7	39.3
BD	16.3	0.7	—	14.5
CB	46.0	41.2	13.8	—

Rows indicate the objective ES being managed for (CP—coastal protection; F—fisheries; BD—biodiversity; CB—carbon storage), while columns index the percent by area which are also priority (are co-benefit) for each other ES.

## Discussion

This is the first attempt to map the provisioning of multiple ES services associated with mangroves for an entire nation, to calculate their associated monetary value, and to use this data to prioritise management of mangroves for different ES objectives. We demonstrate that provisioning of various mangrove ES (coastal protection, fisheries, biodiversity, carbon storage) is spatially variable and, while protection of the highest ranked mangrove areas for any individual ES can be done at fairly low opportunity cost, this will not necessarily ensure high levels of co-benefits for the other ES given limited spatial overlaps in priorities. Prioritisation does however result in much greater co-benefit for most ES than random selection of mangrove sites alone. While mapping of ES at high resolution in both data-rich urban (e.g., [55,56]) and natural environments [57,58] is common, ES evaluations in data-poor regions are often limited to single reserves [59] or are not spatially explicit [60]. We are unaware of any attempts to map the value of mangrove ES services explicitly at the community or patch scale across entire data-poor regions, such as Fiji. Coastal protection and fisheries services in Fiji are inherently local and the transfer of benefits to the local community is directly tangible at these fine scales; more importantly, land use policies are often developed in that space [61,62]. Thus, attributing the actual value of an ES to a particular mangrove area is a potentially more powerful tool for informing mangrove resource managers and those charged with regulating coastal development than simply knowing the aggregate or average value of mangrove ES across a region.

Benefit transfer methods do not provide precise market values for ES, but rather estimate the likely magnitude of approximate ES values [63]. While collecting primary economic and biophysical ES data is costly and time consuming [64], and ultimately beyond the scope of this study, we used data from Fiji where available or from nearby regions with similar economic, cultural, and ecological conditions. The validity of ES maps varies depending on the service in question [65], and while we have strived to make reasonable assumptions and use the best available data, uncertainties remain. Estimating coastal protection necessarily requires knowledge of communities and structures in zones at-risk from coastal flooding (under 10 mamsl), and requires quality elevation, census and built infrastructure data. While such data are available in parts of our study area, they are limited. Outside of Fiji's main cities census enumeration areas (EA) are large and lack detail of where people actually reside. Similarly, vertical errors in available satellite-derived elevation data (SRTM) are larger than the elevation under which we consider properties to be at-risk from coastal flooding (e.g., see [66,67]). Other proxies used to estimate development/population, such as night light intensity [68] or road-network density [69], may exclude benefits to poorer villages—areas with few lights or catalogued streets—but where relative ES benefits may be greater.

Our method used to model wave attenuation (e.g., [40]) and coastal protection values assumed 1 m waves arrive on every mangrove area mapped; in reality, mangrove areas are typically found in regions that experience lower wave heights on a daily basis (e.g., [70,71]). On the other hand, the north-western shorelines of both Viti Levu and Vanua Levu are more exposed to the typical track of passing tropical cyclones than other areas of Fiji [72], indicating perhaps an extra benefit for managing mangroves in those areas for coastal protection services. Benefits of shoreline protection also accrue from maintaining mangrove stands over the longer term; for example, village elders have described shorelines retreating inland 10-15m from where they remember mangroves growing as children, but which have since been cleared [73]. Over long time frames mangroves can provide additional benefits by both stabilising and maintaining sediments with sea level rise (SLR) [74], conditional to the rate of SLR and alterations to sediment delivery [75].

The contributions of mangroves to Fiji's important reef fisheries are not well understood, but the value of reef fisheries in Fiji is thought to be significantly larger than that of the mangrove fishery alone which we have valued here [76]. While mangroves provide an important juvenile habitat for many commercially important reef fishes [77], their ontogenetic dispersal value is difficult to evaluate since predicting the amount of time a mangrove's prop roots are inundated (i.e., maximum tide range in Suva is ~1.8 m), and are thus providing nursery habitat, is hard to quantify [78]. These uncertainties are further compounded by poor knowledge of which species in Fiji are obligate and facultative users of mangrove habitats. We introduce additional uncertainty by estimating that a fishery's values can be reduced by up to 50% in mangroves where catchment run-off pollution is high. Fijian newspapers have documented fish kills in the lower reaches of the Ba, Labasa, and Qawa rivers, typically adjacent to sugar mills, but the actual magnitude of the impact on Fijian mangrove fisheries hasn't been fully quantified despite data showing reduced fish abundances and diversity in lower mangrove-fringed reaches of highly-altered river catchments [32,79].

There is debate in the literature as to whether biodiversity should be seen as an ecosystem service or viewed as outside the ES framework yet underpinning the long term provisioning of all other services [80–82]. Our calculations may reflect that division as biodiversity services are the only ES we have considered that carries no direct market value. However, with tourism projected to contribute nearly 50% of Fiji's gross domestic product (GDP) by 2024 [83], the values of biodiversity and the natural environment are clearly central components of both Fiji's conventional and explicitly nature-based tourism draw [84], and have significant value. Accounting for the economic benefit of individual species' contributions to the value of biodiversity provisioning (for example, local income from bird watching tours) would require a better understanding of how and when species are using mangrove habitat, which would allow for more monetised valuation of the mangrove's biodiversity services. We further note that our method does not account for the irreplaceability value that could be placed on known habitats for endemic species of conservation concern, which if incorporated for locations of documented species might dramatically alter the spatial priorities for biodiversity conservation.

Not all services are equal or fungible (i.e., interchangeable) and this needs to be considered from a priority setting perspective. For example, maintaining certain areas that have high BCR values for carbon may make sense since carbon is fungible—a metric ton of CO_2_ on Veti Levu or Vanua Levu will be the same when it comes to climate regulation benefits and value. But biodiversity and coastal protection services are not fungible from place to place—if only areas with the highest BCR for each ES are protected there may still be losses of important elements of biodiversity (i.e., endemic species). Out of the four ES we have mapped only the value of carbon is fungible on the global scale. Nationally regulated carbon markets are currently not developed in Fiji or the larger Pacific Island region (though some upland forests have been placed on voluntary market schemes), yet the non-monetary benefits of carbon storage and sequestration are the same whether in Fiji or Finland. While the creation of carbon markets in the region would begin the process of providing financial incentive for managing mangroves for their carbon storage value, currently the costs of getting into carbon market schemes (e.g., costs associated with accreditation and certification) are often greater than the financial return of entering into a market at present day low global prices of CO_2_e [85]. It is often assumed that carbon storage and sequestration values will overlap with the provisioning of other ES, such as biodiversity, and will therefore provide a mechanism for protecting these other services which are difficult to monetise. However, similar to other studies [86,87], we show that these ES do not necessarily overlap.

Our methods assume that firewood demand is uniform across all of Fiji and makes no distinction between urban and rural populations' actual dependency on or usage of mangrove wood products. In reality there is likely to be a greater variety of fuel replacement alternatives (e.g., electricity, propane, etc.) available in Fiji's developed areas, and local populations in those areas are likely to have higher incomes, and therefore more options for cooking fuels, besides collecting firewood. The collection of firewood in mangroves is likely a more significant ES in Fiji's more remote villages and on Fiji's remote outer group islands, and subsequently, the opportunity cost of firewood would be relatively higher in those areas. The expectations of the services provided by a local mangrove held by local stakeholders [88] are likely to vary around the Fijian archipelago to a great extent, yet are not accounted for here. Further social and cultural values of ecosystems services are difficult to monetise [56], such as those associated with bequest of fishing heritage or existence values, and have not been incorporated. However, such non-use values of mangroves have been estimated to account for approximately 75% of residents' perceived value of mangroves in American Samoa [89].

### Implications for Policy Makers

Spatial mapping that indicates geographical distinctions between priority mangroves for provisioning of different ES can help decision-makers direct funding for mangrove management from various sources to localities that best meet the funding objectives [90]. For example, financing for disaster risk reduction and climate adaptation (e.g., from the Green Climate Fund) can be directed toward mangrove areas with the highest coastal protection services, while biodiversity funds (e.g. from the Global Environment Facility, GEF) can be directed towards areas with the highest potential to conserve species. Presently in Fiji, partly because there has been no national-scale accounting of ES values, GEF funding for biodiversity that was bundled with funding for climate adaptation and sustainable land management has been allocated to some of the most degraded habitats in the country (S. Jupiter, personal communication), resulting in a major lost opportunity for effective biodiversity financing and conservation.

Secondly, our approach might be used for coarse level designation of no-go zones for development based on their national significance for ES provisioning. A preliminary zoning scheme in Fiji’s first Mangrove Management Plan included resource and nature reserve designations, where development should not be allowed, within three of Fiji’s largest mangrove deltas [45]. The purpose of the original zoning scheme was to provide government regulators with a decision-making framework for evaluating development applications and special licensing (e.g, for mangrove timber harvesting). However, these zones were not based on any rigorous assessment of ES values and were never implemented. Our more comprehensive national-scale assessment might allow for the selection of the highest priority areas for each ES where development and extractive activities are not allowed, noting that further studies might be warranted to additionally assess other mangrove ES values (e.g., recreation, bequest/existence, water purification, nutrient cycling, cultural uses) identified as locally important in Fiji's updated draft management plan, endorsed by the national Mangrove Management Committee [91].

Yet decision-makers must be mindful of equity issues when selecting areas to zone for reserves versus development. Spatial priorities will depend on what is being valued and whose objectives are being met, resulting in trade-offs where there are competing objectives and values [92,93]. For example, our estimation of the BCR of mangrove coastal protection services accounted for the replacement costs of infrastructure, which directed priorities towards mangroves near urban areas with dense networks of expensive infrastructure (Fig 4). However, residents of rural villages and settlements may in fact be more physically vulnerable to the impacts of severe storm surge events [94]. Thus, we suggest that caution should be taken to ensure that the value of local mangrove services for rural, poorer areas are factored into decision-making, regardless of how high those mangrove areas rank on a national scale for ES provisioning.

## Supporting Information

S1 Fig Examples of broken up large mangrove areas. Mangroves around the Ba river delta (a), the Tuva river delta (b), and the Rewa river delta, on all on Viti Levu. Differing ordination of fill lines indicate how larger mangrove areas were broken into smaller planning units.
(TIF)

S2 Fig Correlation plots of coastal protection inputs. hh_pu = households in PU; bldg_risk = buildings at risk in PU; bldg_cost = building cost in PU; t_rd_rpr = total road repair cost in PU; t_tram_rpr = total tramway repair cost in PU; Pvwb_wr_wt = total present coastal protection benefit in PU; L = total lost lease payments in PU; T = total cost of firewood replacement (cooking fuel) in PU; t_cost = total opportunity cost in PU; b_c = benefit-cost-ratio in PU. Numbers above the diagonal are the value of the correlation while stars are the result of the correlation test (where '***' = *p* < 0.001, '**' = *p* < 0.01, '*' = *p* < 0.05, and '.' = *p* < 0.1). Below the diagonal are bivariate scatterplots with fitted lines.
(TIF)

S3 Fig Correlation plot of fisheries service inputs. hh_pu = households in PU; dst_mrk = travel distance to market; PV_con = present value of subsistence fishery in PU; PV_mrkt = present value of market fishery; *c* = percent coral cover within 10 km; L = total lost lease payments in PU; T = total cost of firewood replacement (cooking fuel) in PU; t_ben = total fisheries benefit in PU; t_cost = total opportunity cost in PU; b_c = benefit-cost-ratio in PU. Numbers above the diagonal are the value of the correlation while stars are the result of the correlation test (where '***' = *p* < 0.001, '**' = *p* < 0.01, '*' = *p* < 0.05, and '.' = *p* < 0.1). Below the diagonal are bivariate scatterplots with fitted lines.
(TIF)

S4 Fig Correlation plot of biodiversity inputs. reef_area = total coral reef area within 10 km; hh_pu = households in PU; *S* = total predicted number of species present in PU; L = total lost lease payments in PU; T = total cost of firewood replacement (cooking fuel) in PU; F = total subsistence and market fisheries value in PU; t_cost = total opportunity cost in PU; b_c = benefit-cost-ratio in PU. Numbers above the diagonal are the value of the correlation while stars are the result of the correlation test (where '***' = *p* < 0.001, '**' = *p* < 0.01, '*' = *p* < 0.05, and '.' = *p* < 0.1). Below the diagonal are bivariate scatterplots with fitted lines.
(TIF)

S5 Fig Correlation plot of carbon storage inputs. hh_pu = households in PU; L = total lost lease payments in PU; T = total cost of firewood replacement (cooking fuel) in PU; t_cost = total opportunity cost in PU; b_c = benefit-cost-ratio in PU. Numbers above the diagonal are the value of the correlation while stars are the result of the correlation test (where '***' = *p* < 0.001, '**' = *p* < 0.01, '*' = *p* < 0.05, and '.' = *p* < 0.1). Below the diagonal are bivariate scatterplots with fitted lines.
(TIF)

S6 Fig Coastal protection net benefit values. (TIF)

S7 Fig Fisheries net benefit values. (TIF)

S8 Fig Biodiversity net benefit. Net benefit is measured as the estimated number of species present in the mangrove.
(TIF)

S9 Fig Carbon storage net benefit values. (TIF)

S10 Fig Forest cover (green area areas) among Fiji's larger river catchments (delineated by black lines; upper panel) and modelled run-off pollution per catchment (bottom panel). (TIF)

S1 Table Spatial data on layers, sources and data processing. (PDF)

S2 Table Fish market values (March 2015). (PDF)

S3 Table Building replacement values by District (*Tikina*) and Province. (PDF)
