# Supplementary figures and images for: Prioritising Mangrove Ecosystem Services Results in Spatially Variable Management Priorities
Source: PLoS One. 2016 Mar 23;11(3):e0151992. doi: 10.1371/journal.pone.0151992 (PMC4805192; doi:10.1371/journal.pone.0151992)

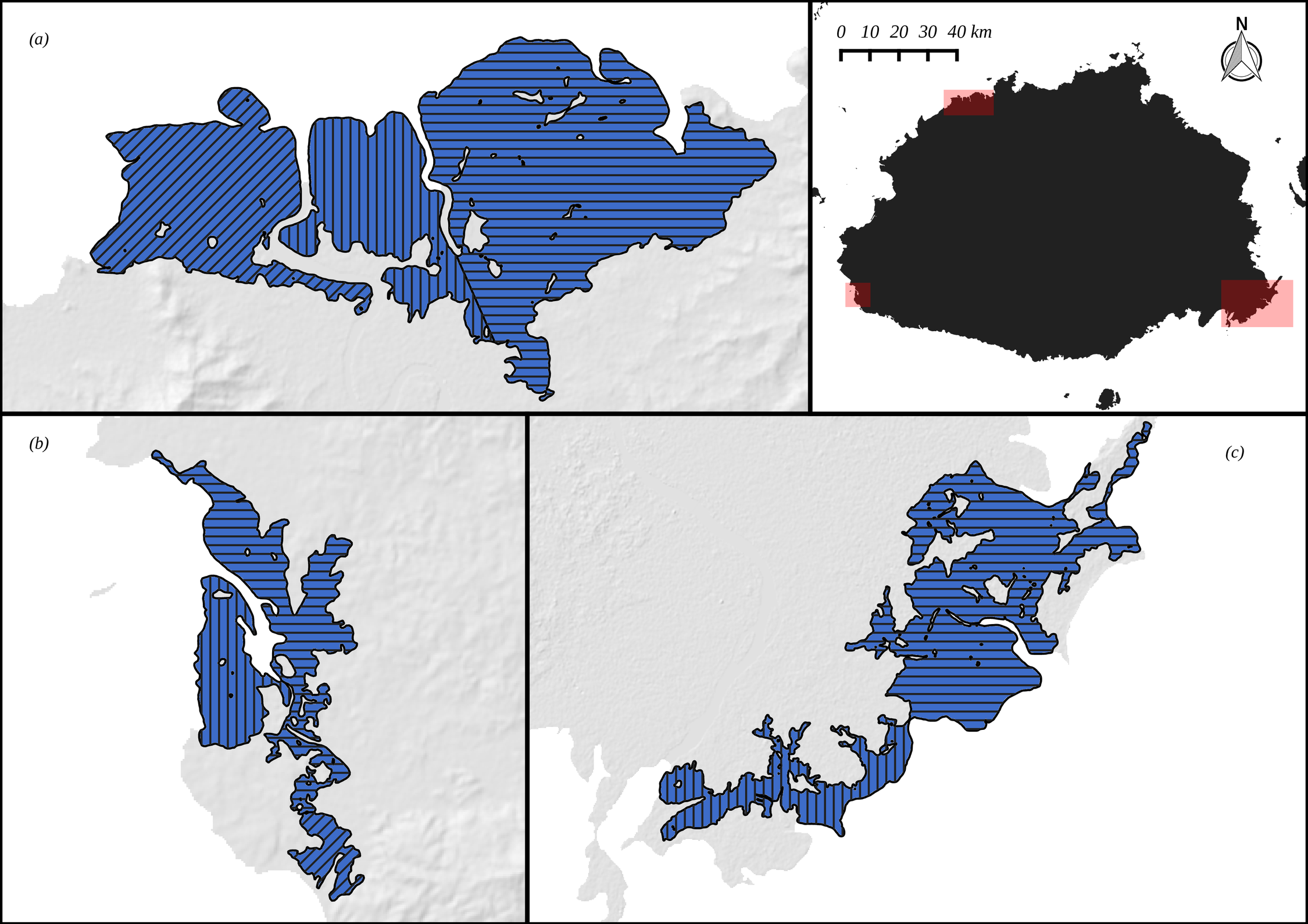

Supplement: S1 Fig — Mangroves around the Ba river delta (a), the Tuva river delta (b), and the Rewa river delta, on all on Viti Levu. Differing ordination of fill lines indicate how larger mangrove areas were broken into smaller planning units. (TIF) [file pone.0151992.s001.tif]

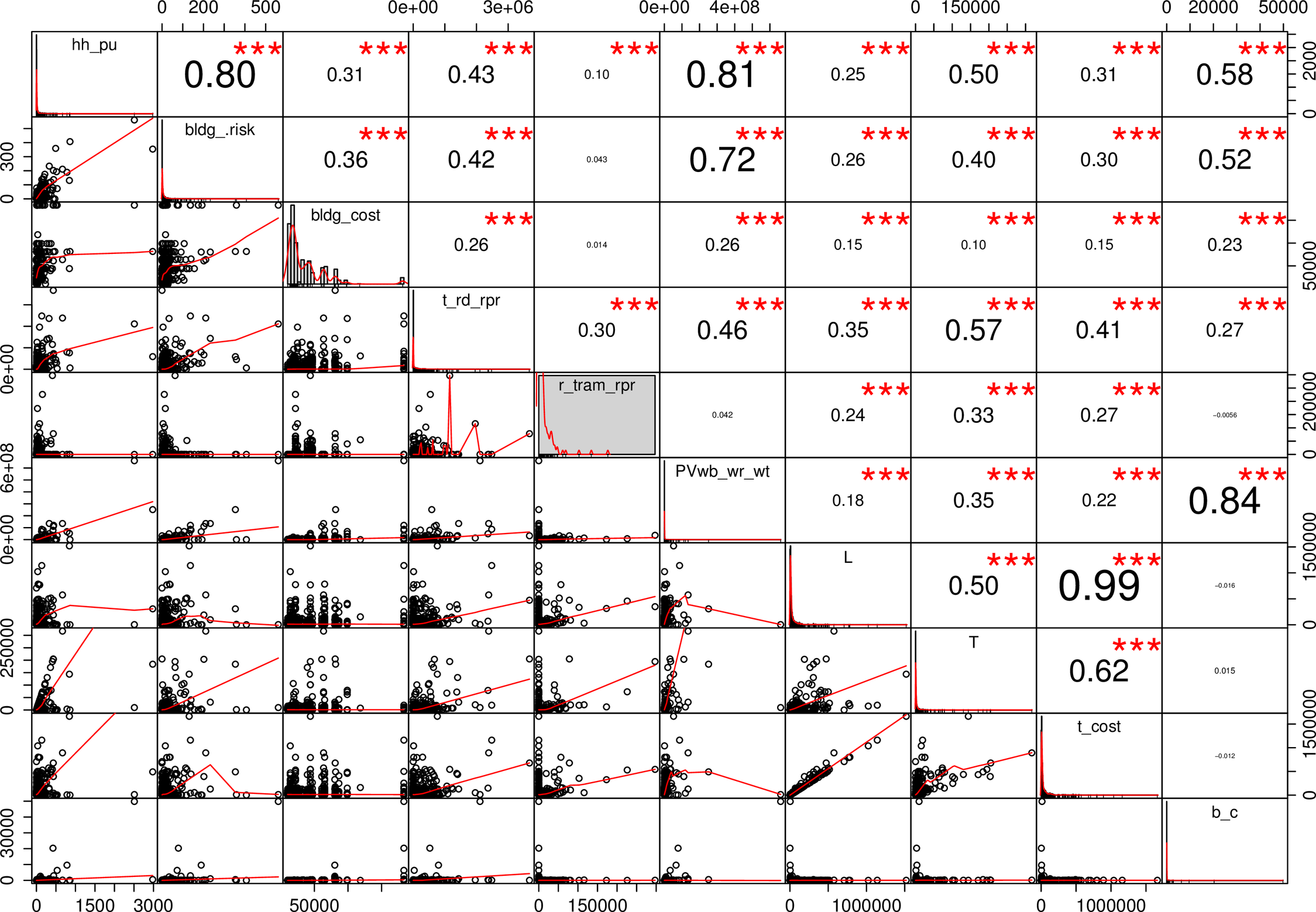

Supplement: S2 Fig — hh_pu = households in PU; bldg_risk = buildings at risk in PU; bldg_cost = building cost in PU; t_rd_rpr = total road repair cost in PU; t_tram_rpr = total tramway repair cost in PU; Pvwb_wr_wt = total present coastal protection benefit in PU; L = total lost lease payments in PU; T = total cost of firewood replacement (cooking fuel) in PU; t_cost = total opportunity cost in PU; b_c = benefit-cost-ratio in PU. Numbers above the diagonal are the value of the correlation while stars are the result of the correlation test (where '***' = p < 0.001, '**' = p < 0.01, '*' = p < 0.05, and '.' = p < 0.1). Below the diagonal are bivariate scatterplots with fitted lines. (TIF) [file pone.0151992.s002.tif]

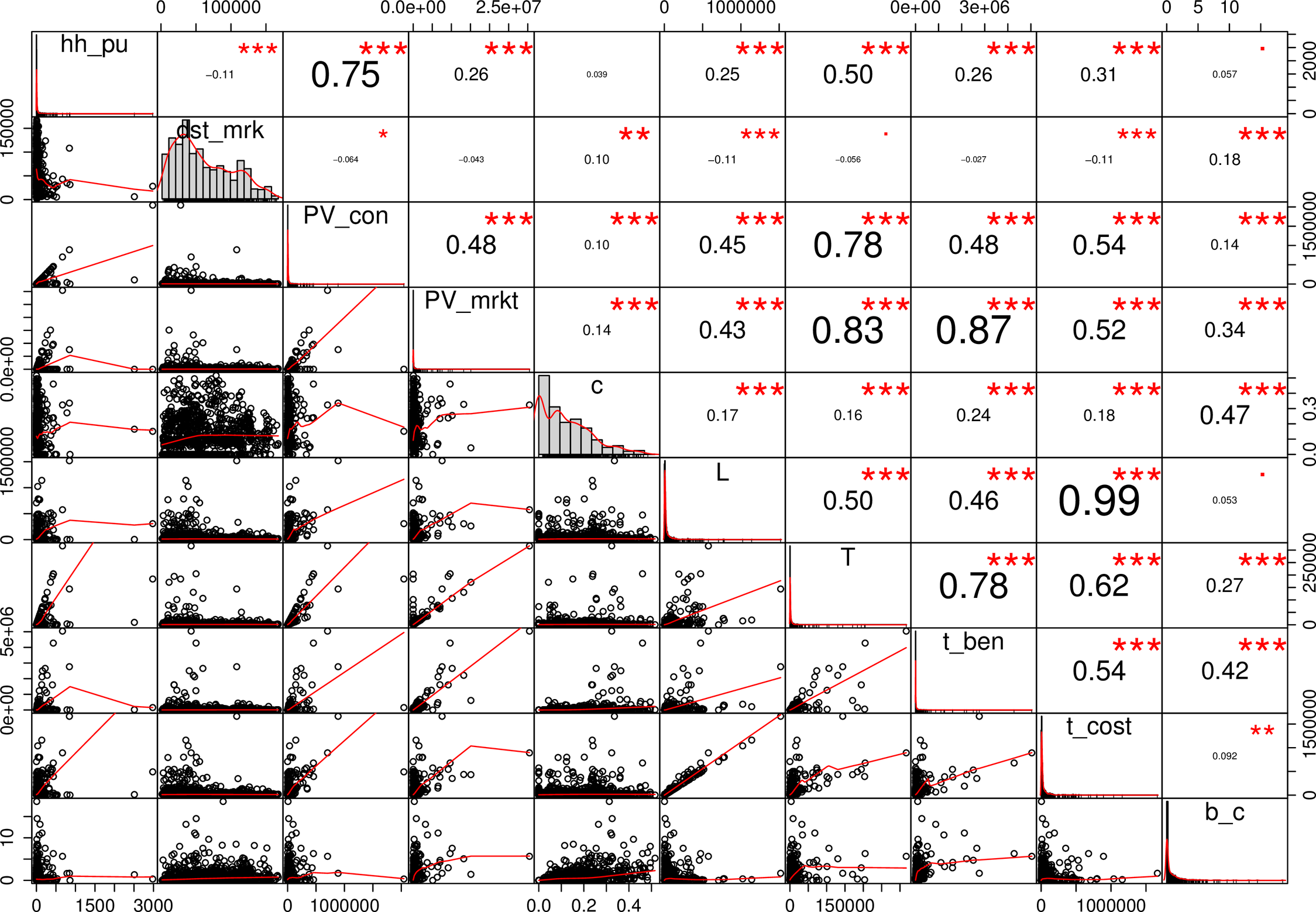

Supplement: S3 Fig — hh_pu = households in PU; dst_mrk = travel distance to market; PV_con = present value of subsistence fishery in PU; PV_mrkt = present value of market fishery; c = percent coral cover within 10 km; L = total lost lease payments in PU; T = total cost of firewood replacement (cooking fuel) in PU; t_ben = total fisheries benefit in PU; t_cost = total opportunity cost in PU; b_c = benefit-cost-ratio in PU. Numbers above the diagonal are the value of the correlation while stars are the result of the correlation test (where '***' = p < 0.001, '**' = p < 0.01, '*' = p < 0.05, and '.' = p < 0.1). Below the diagonal are bivariate scatterplots with fitted lines. (TIF) [file pone.0151992.s003.tif]

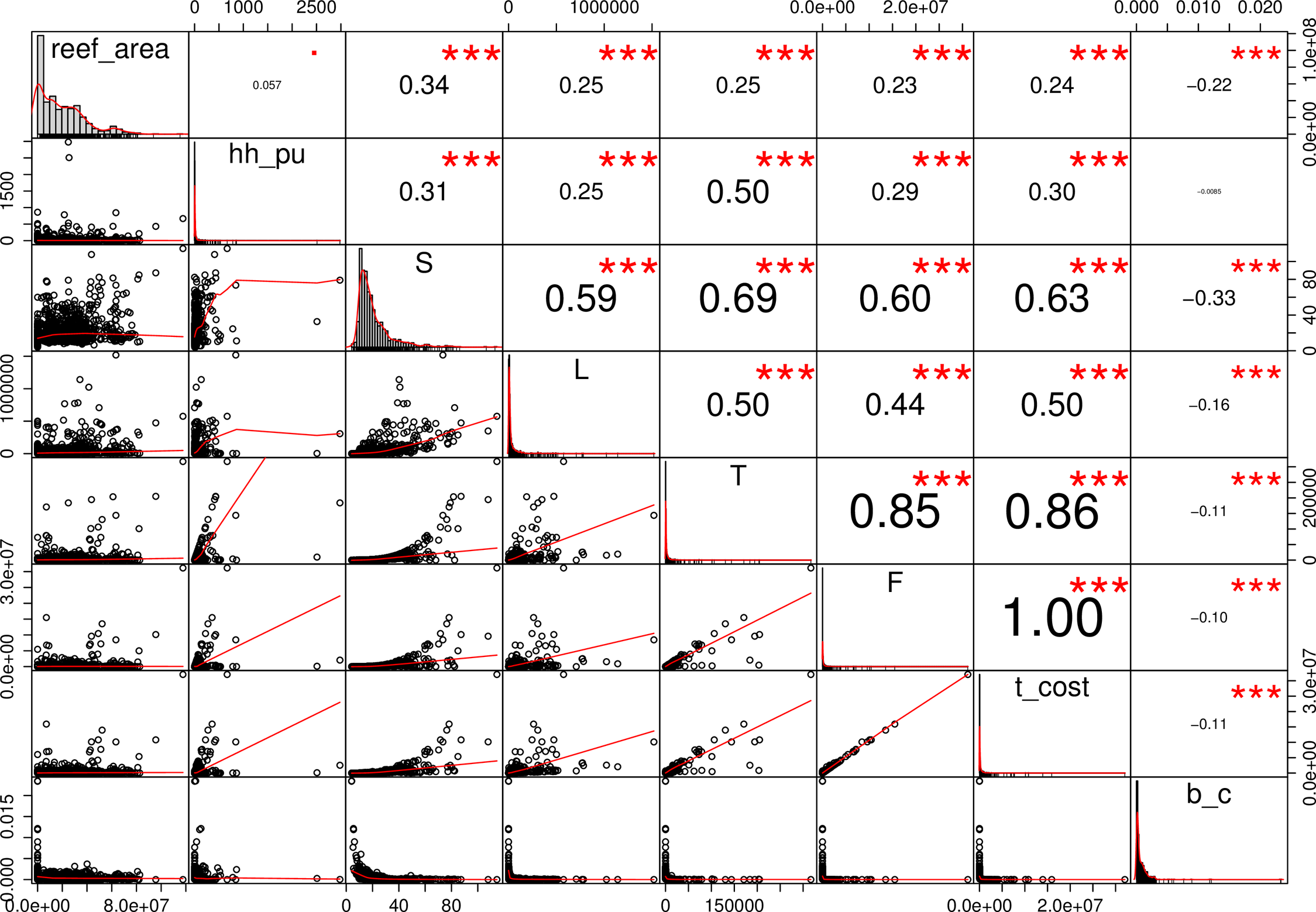

Supplement: S4 Fig — reef_area = total coral reef area within 10 km; hh_pu = households in PU; S = total predicted number of species present in PU; L = total lost lease payments in PU; T = total cost of firewood replacement (cooking fuel) in PU; F = total subsistence and market fisheries value in PU; t_cost = total opportunity cost in PU; b_c = benefit-cost-ratio in PU. Numbers above the diagonal are the value of the correlation while stars are the result of the correlation test (where '***' = p < 0.001, '**' = p < 0.01, '*' = p < 0.05, and '.' = p < 0.1). Below the diagonal are bivariate scatterplots with fitted lines. (TIF) [file pone.0151992.s004.tif]

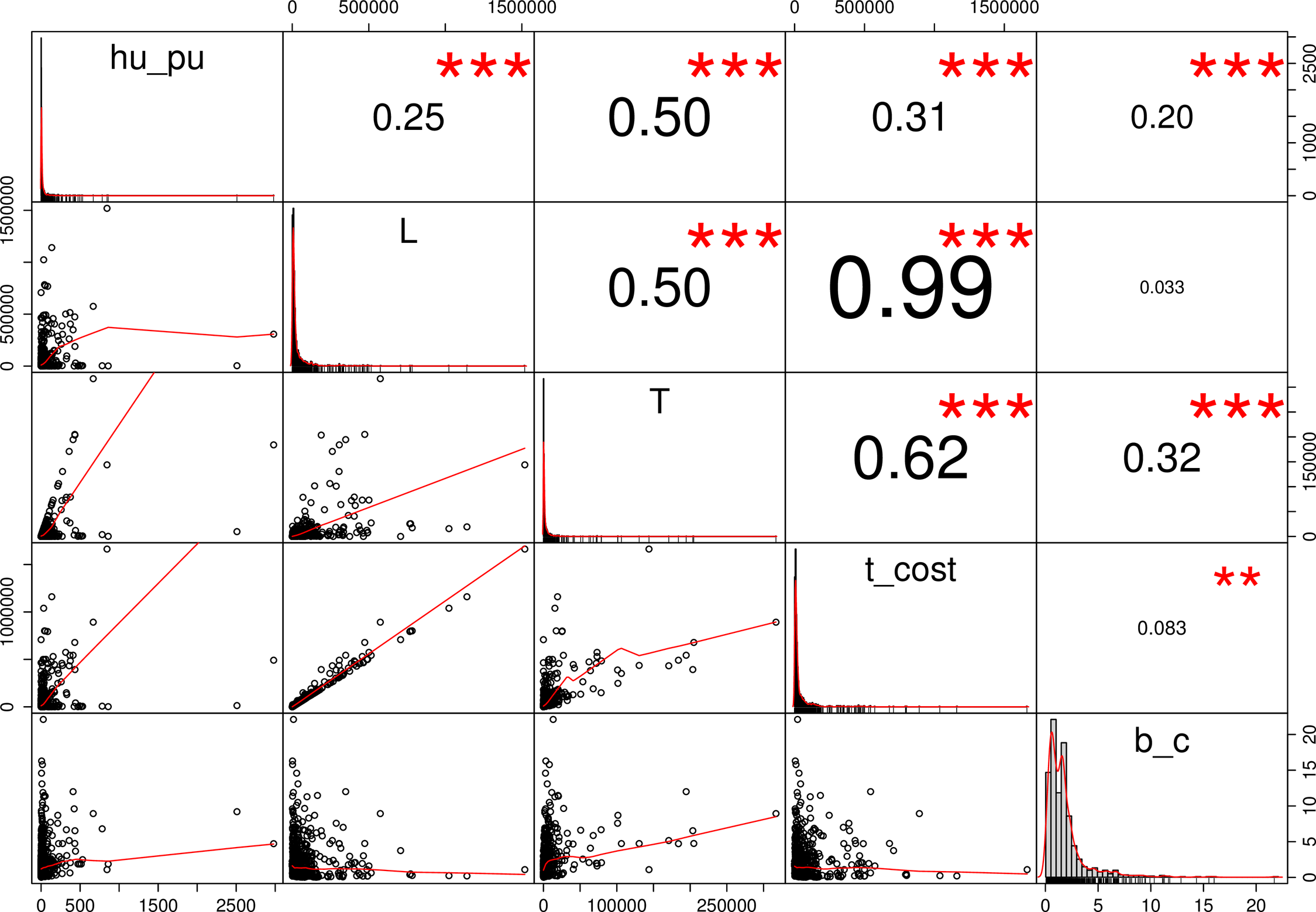

Supplement: S5 Fig — hh_pu = households in PU; L = total lost lease payments in PU; T = total cost of firewood replacement (cooking fuel) in PU; t_cost = total opportunity cost in PU; b_c = benefit-cost-ratio in PU. Numbers above the diagonal are the value of the correlation while stars are the result of the correlation test (where '***' = p < 0.001, '**' = p < 0.01, '*' = p < 0.05, and '.' = p < 0.1). Below the diagonal are bivariate scatterplots with fitted lines. (TIF) [file pone.0151992.s005.tif]

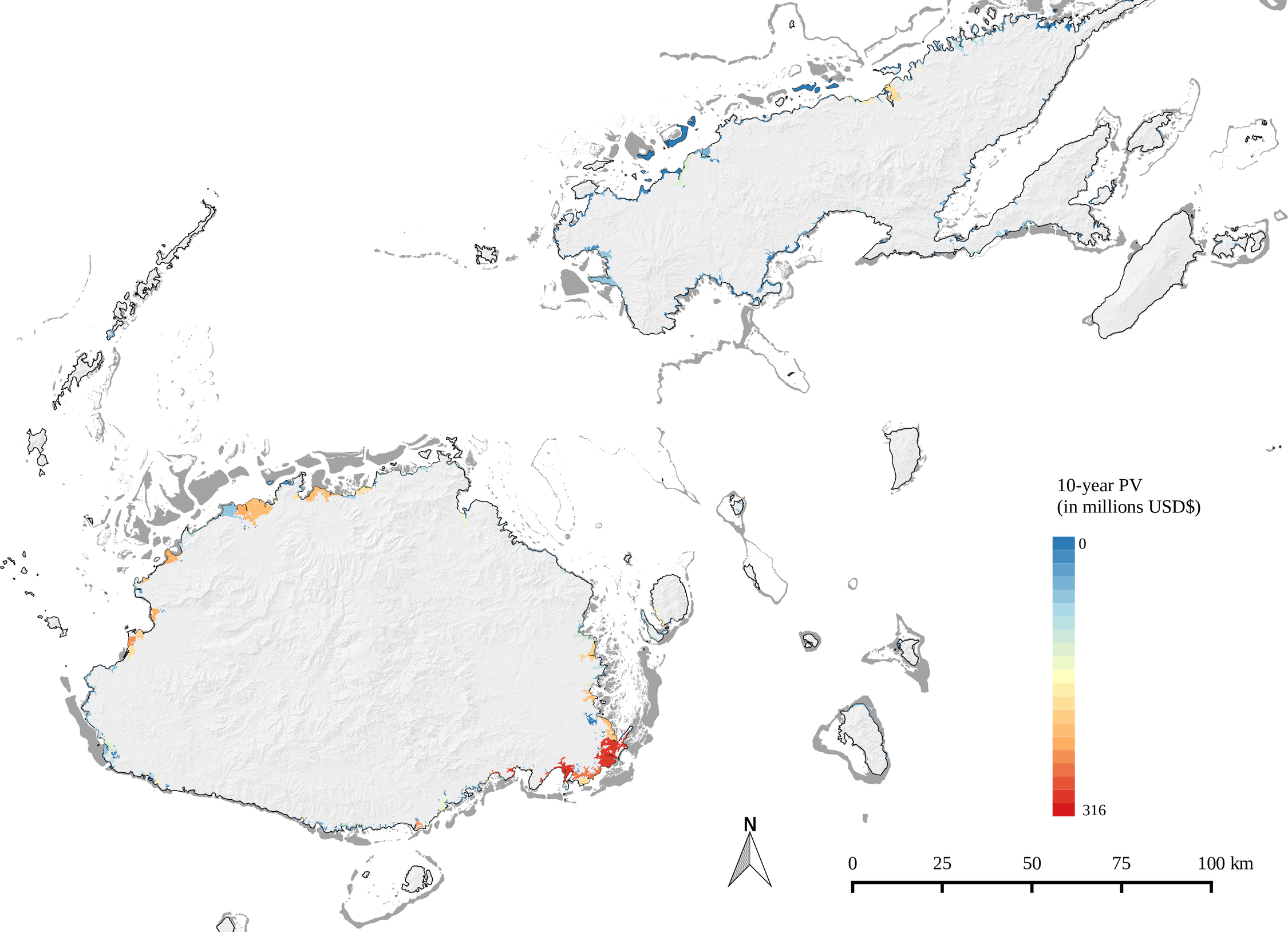

Supplement: S6 Fig — (TIF) [file pone.0151992.s006.tif]

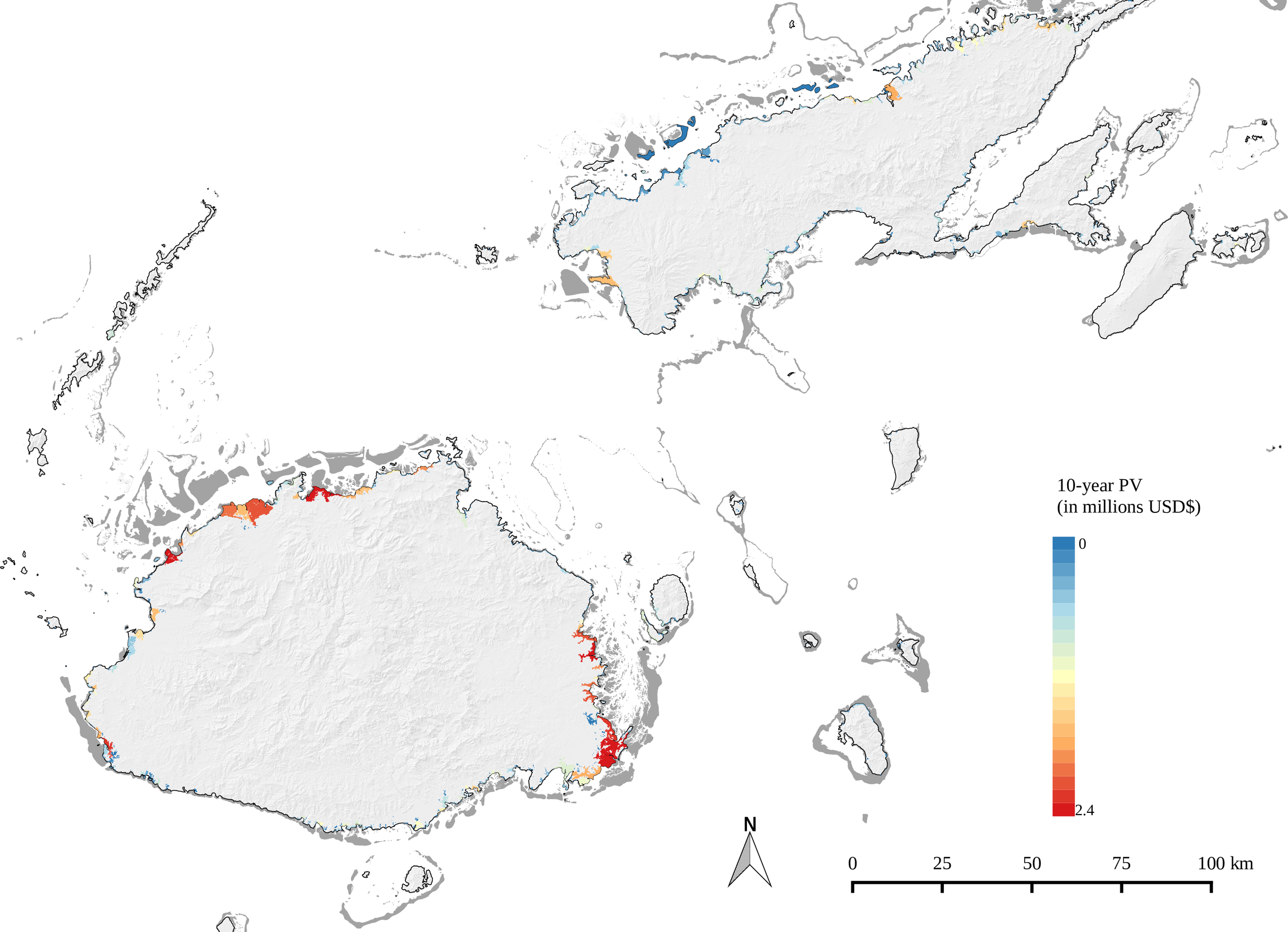

Supplement: S7 Fig — (TIF) [file pone.0151992.s007.tif]

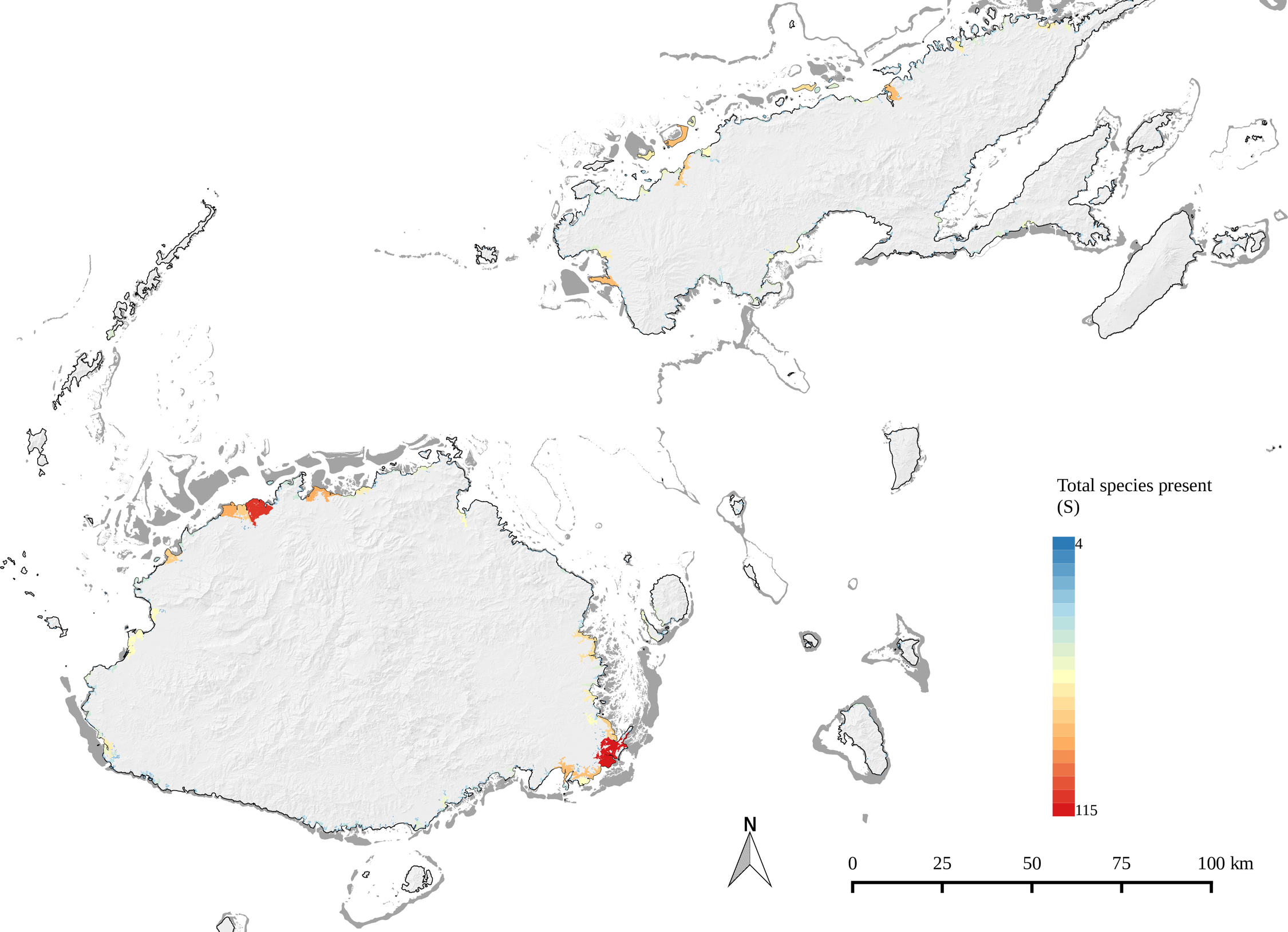

Supplement: S8 Fig — Net benefit is measured as the estimated number of species present in the mangrove. (TIF) [file pone.0151992.s008.tif]

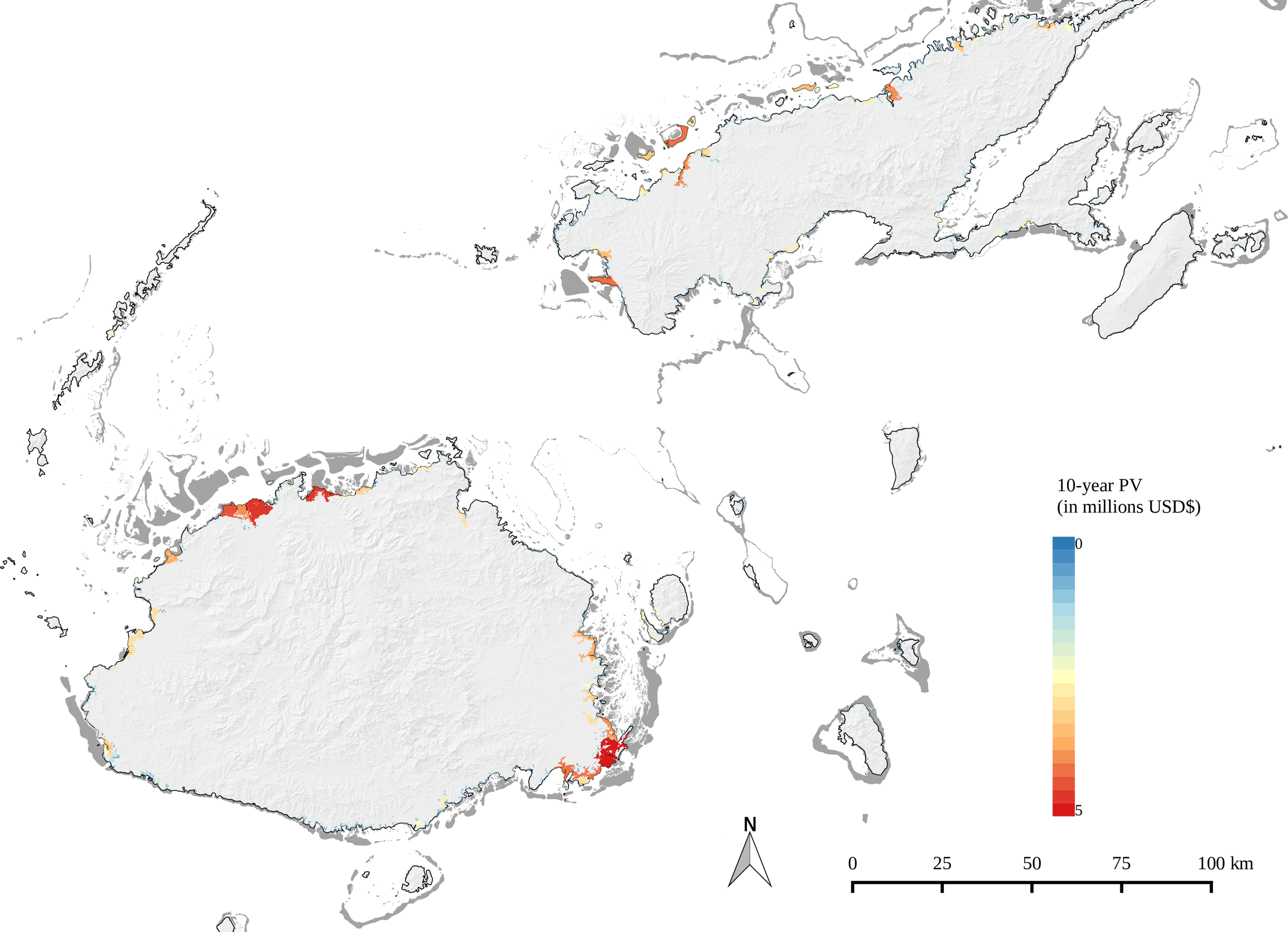

Supplement: S9 Fig — (TIF) [file pone.0151992.s009.tif]

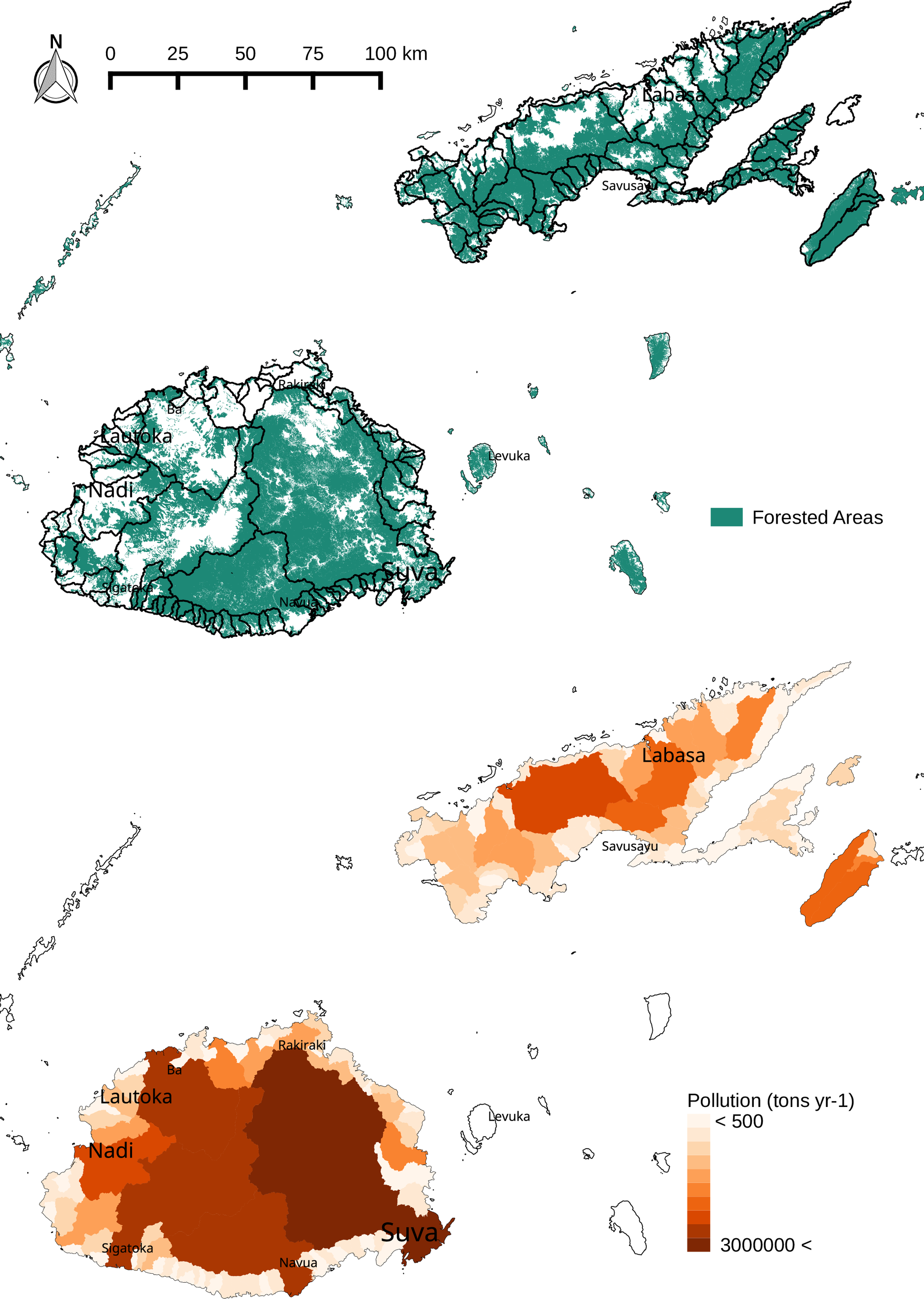

Supplement: S10 Fig — (TIF) [file pone.0151992.s010.tif]
