# Supplementary material for: Prioritising Mangrove Ecosystem Services Results in Spatially Variable Management Priorities
Source: PLoS One. 2016 Mar 23;11(3):e0151992. doi: 10.1371/journal.pone.0151992 (PMC4805192; doi:10.1371/journal.pone.0151992)
Supplement: S1 Table — (PDF) [file pone.0151992.s011.pdf]

S1 Table. Spatial data on layers, sources and data processing

Layer name	Mapped feature	Source	Dataset details	Processing undertaken (software used)	Processing details
Coastline	Coastline	United States Geological Survey (USGS)	Derived from Shuttle Radar Topography Mission (SRTM) global 1 arc-second digital elevation model (DEM; ~30 m horizontal resolution)	Extracted areas above sea level (e.g., 0 metres above mean sea level (masml)) and polygonised (QGIS 2.10)	Pixels in the DEM above 0 mamsl were selected using a raster calculator, and converted to simplified polygons. Some manual digitizing was required where the coastline deviated dramatically from visual comparison with high resolution satellite imagery, or where changes in the coastline due to development, etc., have occurred since the DEM was developed.
Mangroves	Mangrove	1 | Fiji Department of Forestry, Ministry of Fisheries and Forests<br>2 | Hamilton and Casey (2014)	1 | Digitized for main Fiji islands (Viti Levu, Vnaua Levu, and Taveuni only) from 2001 Landsat ETM+ data<br>2 | Multiple dataset ‘Big Data’ fusion approach – see source. Raster attribute is m^2 of mangrove per pixel.	Converted raster areas in (2) to polygons and merged with (1) to create a single layer (QGIS 2.10)	Raster cells from (2) with values greater than 225 (e.g., greater than 25% mangrove) were selected with a raster calculator and converted to polygons. The new combined layer was clipped to the coastline layer to ensure topological continuity.
Reefs	1 | Fringing reefs<br>2 | Non-fringing reefs	Fiji Department of Lands, Ministry of Lands and Mineral Resources	Digitized from aerial photographs captured in 1994 and 1996	Merged fringing reef (1) and non-fringing reef (2) layers into a single combined reef layer (QGIS 2.10)	Erased overlap with coastline by overwriting reefs with coastline layer.
Habitable area	Liveable / build-able areas	USGS	Derived from SRTM global 1 arc-second DEM (~30 m horizontal resolution)	Created a slope raster; converted areas with slopes under 20% and under 100 mamsl into polygons (QGIS 2.10)	Selected cells slope raster values less 20% and elevation values less than 100m with a raster calculator; converted the output to polygons.
Ocean within 10km	Connected ocean area	NA	NA	Cost Distance with a barrier (ArcGIS 10.2)	Created a cost raster with ocean area cost values set to 1 and land area cost values set to NA, in essence making a barrier to travel though land areas; pixels within 10 km travel distance (limited to transit by ocean areas only) were selected and converted to polygons.

Enumeration Areas(w/ population data)	Population	Fiji Bureau of Statistics	1 | Population enumeration area (EA) shapefiles<br>2 | 2007 census data	Join of EA shapefile with 2007 census data. Calculated population density (QGIS 2.10)	The population density of each EA was derived by intersecting each EA with the habitable area layer, calculating the density of the census population over that habitable area assuming a uniform spatial distribution of the population.
Forest Cover	Land cover	Pacific Catastrophe Risk Assessment and Financing Initiative (PCRAFI)	NA	Export of forest layers (QGIS 2.10)	Selected polygons with land cover attributes of ‘Forest’ or ‘Scattered Forest’. Derived the % of forest cover for each catchment by intersecting forest and catchments layers.
Pollution per catchment	Modelled pollution estimates per watershed	Klein et al. (2012)	Derived from Klein et al. (2012) who estimated maximum pollution from a catchment assuming all forest is cleared; we modify those estimates by assuming total pollution is proportional to forest cover in a watershed.	New field calculation (QGIS 2.10)	Multiplied total modelled pollution per catchment by the % forest cover in each catchment.
Villages	Village areas	Geospatial Information Management Unit, Fiji Department of Lands, Ministry of Lands and Mineral Resources	Polygons of village boundaries	None	None
Buildings	Building features	PCRAFI	Building footprint polygons; only in area around main cities.	Spatial join of attributes (QGIS 2.10)	Joined attributes from verified layer of buildings (points) to the polygon layer of buildings. The verified layer is also not complete.
Roadways	Roadway networks	Geospatial Information Management Unit, Department of Lands, Ministry of Lands and Mineral Resources	Comprehensive road network layer with breakdown by sealed and unsealed road sections	None	

Tramlines	Sugarcane tramline network	Geospatial Information Management Unit, Department of Lands, Ministry of Lands and Mineral Resources	NA		
Distance to Market Network	Nearest major fish market by distance	NA	Polyline network layer	Connect mangroves to road network and shortest route to nearest fish market (GRASS GIS 6.4)	Mangrove centroids were connected to roads within 10 km using v.net function; the shortest distance to fish market over the road network was found using the v.net.distance function. Not all mangroves could reach a market over the road network.
Planning units (PUs)	Planning Unit	NA	NA	Cost Allocation (ArcGIS 10.2)	The cost allocation tool was set to allocate areas to their nearest source mangrove polygon within a distance of 500m. Where mangroves are within 1 km of each other, the PU boundary is equidistant between the two; areas were converted to polygons.
Mangrove depth	Wave attenuation	NA		Cost Distance with barrier (ArcGIS 10.2)	The coastline layer was rasterised; mangrove areas were given a value of 1 and non-mangrove areas given a value of NA, creating a barrier. The distance to ocean was calculated as the nearest distance of a cell to ocean through mangrove, e.g., not a straight line. Not all mangroves had direct connection to the ocean and thus couldn't be included.
Mangrove depth at points where wave protection is provisioned	Coastal protection	NA	Derived from overlap of the edge of mangrove areas and infrastructure of interest, .e.g., buildings, roads and tramlines	Point sampling of mangrove depth (QGIS 2.10)	Created a 75 m buffer around buildings and infrastructure and created points where the buffer intersected with the landward edge of a mangrove; the depth of the raster at those points was extracted and the mean value for each mangrove was calculated from the mangrove depth raster.
Population per PU	Estimated Population	NA	Derived from:<br>- PU layer<br>- Habitable area layer<br>- Population enumeration area	Polygon intersections (QGIS 2.10)	The area of the PU was multiplied by the population density of the EA in which it was located. Where a PU overlapped more than one EA, the estimated population within the portion of each EA falling within the PU was first calculated, and then estimated populations from each EA portion were summed.

PU Population at risk	Estimated population	NA	Derived from:<br>- Population per PU<br>- Sub 10 mamsl layer	Polygon Intersections (QGIS 2.10)	The estimated population of a PU was multiplied by the % of the PU under 10 mamsl; this assumes equal population density in the PU. Where a PU overlapped more than one EA (as above) the estimated population of the portion of each EA under 10 masml within a PU was first calculated, and then summed.
Sub 10m risk zone	Elevation	USGS	Derived from SRTM global 1 arc-second DEM (~30 m horizontal resolution; Note: < 16 absolute vertical height error	Extract areas under 10masml (QGIS 2.10)	Used a raster calculator to select areas with an elevation under 10 mamsl; converted the layer to polygons.
Buildings at risk	Buildings in sub-10m zone	PCRAFI		Polygon intersections (QGIS 2.10),<br>Linear model (R 3.2.1)	The number of residential buildings within 100m of the landward mangrove edge was derived for urban areas where some buildings footprints have been polygonised and are available. Outside of those areas we modeled the estimated number of residential buildings as a function of village area by extracting buildings polygons within 'village' areas nearby to urban centers where shapefile data are available. Assuming that all villages in Fiji have similar densities and building types, we used the coefficients of that model to estimate the number of residential buildings in other village polygons.
