# Supplementary material for: Prioritising Mangrove Ecosystem Services Results in Spatially Variable Management Priorities
Source: PLoS One. 2016 Mar 23;11(3):e0151992. doi: 10.1371/journal.pone.0151992 (PMC4805192; doi:10.1371/journal.pone.0151992)
Supplement: S2 Table — (PDF) [file pone.0151992.s012.pdf]

S3 Table. Fish market values (March 2015)

Market Name	Fish Grade	Min Price	Max. Price	Average Price	Notes
Ba	A	$10.00	$15.00		Coral trout, some emperor species, most groupers, Spanish mackerel
Ba	B	$7.00	$8.00		Red snapper and some other emperors
Ba	C	$2.00	$2.50	$10.09	Trevally, Indian mackerel, silasila
Ba	D	$1.50	$1.70		Garfish
Ba	M	$9.76	$43.39		$50-80 per bundle on roadside, unknown weight
Lautoka	A	$25.00	$30.00		Camouflage grouper, spangled emperor, pacific yellow-tail emperor, vermiculated spinefoot
Lautoka	B	$15.00	$18.00		Blue tail mullet, mangrove snapper, mangrove jack
Lautoka	C	$15.00	$30.00	$23.38	Giant trevally, scribbled rabbitfish, parrotfish
Lautoka	D	$15.00			
Lautoka	M	$24.41	$37.97		Per bundle, unknown weight
Sigatoka	A	$30.00	$35.00		
Sigatoka	B	$20.00	$25.00		
Sigatoka	C	$15.00	$18.00	$21.70	
Sigatoka	D	$5.00	$12.00		
Sigatoka	M	$24.41	$32.55		per bundle
Suva	A	$25.00	$30.00		
Suva	B	$16.00	$20.00		
Suva	C	$12.00	$15.00	$20.55	
Suva	D	$10.00			
Suva	M	$13.56	$43.39		per bundle
Labasa				$18.93	Average price of all markets data was collected at
Other				$5.04	Assumed to be half the value of the lowest market prices, found at Ba

: where fish grades run from A (highest quality) to D (lowest value), and M is the price of Qari (mudcrabs, Scylla serrata)
