# Supplementary material for: Prioritising Mangrove Ecosystem Services Results in Spatially Variable Management Priorities
Source: PLoS One. 2016 Mar 23;11(3):e0151992. doi: 10.1371/journal.pone.0151992 (PMC4805192; doi:10.1371/journal.pone.0151992)
Supplement: S3 Table — (PDF) [file pone.0151992.s013.pdf]

S12 Table. Building replacement values by District (*Tikina*) and Province:

Tikina	Province	Replacement Value (USD$)	Tikina	Province	Replacement Value (USD$)
Ba	Ba	21047.38	Rabi	Cakaudrove	7128.44
Baravi	Nadroga	16228.96	Rakiraki	Ra	22493.25
Batiki	Lomaiviti	9229.45	Rewa	Rewa	29160.48
Bau	Tailevu	30461.31	Rewa	Tailevu	29160.48
Bua	Bua	4957.90	Saivou	Ra	9351.56
Cakaudrove	Cakaudrove	6605.02	Saqani	Cakaudrove	7217.76
Cuvu	Nadroga	11918.02	Sasa	Macuata	6311.98
Dogotuki	Macuata	8606.92	Serua	Serua	31228.62
Gau	Lomaiviti	11207.10	Suva	Rewa	87609.52
Koro	Lomaiviti	9566.45	Tavua	Ba	38733.15
Labasa	Macuata	18905.72	Tavuki	Kadavu	9166.93
Lami	Rewa	38447.49	Tunuloa	Cakaudrove	6651.19
Lomai_OtherIs	Lomaiviti	12446.64	Vaturova	Cakaudrove	6813.60
Lomaloma	Lau	7278.35	Veivatuloa	Namosi	6774.47
Macuata	Macuata	9041.08	Verata	Tailevu	8891.46
Malolo	Nadroga	11921.84	Vuda	Ba	21084.80
Malomalo	Nadroga	10481.28	Vuya	Bua	7624.58
Matuku	Lau	9193.84	Wailevu	Bua	9777.17
Moala	Lau	11611.49	Wainikeli	Cakaudrove	8753.47
Mualevu	Lau	19480.78	Wainunu	Bua	4875.03
Naceva	Kadavu	8638.95	Yasawa	Ba	13570.74
Nadi	Ba	47303.77			
Nairai	Lomaiviti	8244.75			
Naitasiri	Naitasiri	39027.23			
Nakasaleka	Kadavu	7154.87			
Nakelo	Tailevu	9251.20			
Nakorotubu	Ra	8574.67			
Nasavusavu	Cakaudrove	16350.49			
Nasigatoka	Nadroga	56642.42			
Naviti	Ba	12079.88			
Noco	Rewa	15438.64			
Nuku	Serua	15131.28			
Ovalau	Lomaiviti	41363.78			
